# Targeting glucosylceramide synthase induces antiproliferative and proapoptotic effects in osimertinib-resistant NSCLC cell models

**DOI:** 10.1038/s41598-024-57028-8

**Published:** 2024-03-18

**Authors:** Silvia La Monica, Federica Vacondio, Kamal Eltayeb, Alessio Lodola, Francesco Volta, Martina Viglioli, Francesca Ferlenghi, Francesca Galvani, Maricla Galetti, Mara Bonelli, Claudia Fumarola, Andrea Cavazzoni, Lisa Flammini, Michela Verzè, Roberta Minari, Pier Giorgio Petronini, Marcello Tiseo, Marco Mor, Roberta Alfieri

**Affiliations:** 1 Department of Medicine and Surgery, University of Parma, 43126 Parma, Italy; 2 Department of Food and Drug, University of Parma, 43124 Parma, Italy; 3 Department of Occupational and Environmental Medicine, Epidemiology and Hygiene, INAIL-Italian Workers’ Compensation Authority, 00078 Monte Porzio Catone, Rome, Italy; 4 Medical Oncology Unit, University Hospital of Parma, 43126 Parma, Italy

**Keywords:** Cancer therapy, Non-small-cell lung cancer

## Abstract

The EGFR tyrosine kinase inhibitor osimertinib has been approved for the first-line treatment of *EGFR*-mutated Non-Small Cell Lung Cancer (NSCLC) patients. Despite its efficacy, patients develop resistance. Mechanisms of resistance are heterogeneous and not fully understood, and their characterization is essential to find new strategies to overcome resistance. Ceramides are well-known regulators of apoptosis and are converted into glucosylceramides (GlcCer) by glucosylceramide synthase (GCS). A higher content of GlcCers was observed in lung pleural effusions from NSCLC patients and their role in osimertinib-resistance has not been documented. The aim of this study was to determine the therapeutic potential of inhibiting GCS in NSCLC *EGFR*-mutant models resistant to osimertinib in vitro and in vivo. Lipidomic analysis showed a significant increase in the intracellular levels of glycosylceramides, including GlcCers in osimertinib resistant clones compared to sensitive cells. In resistant cells, the GCS inhibitor PDMP caused cell cycle arrest, inhibition of 2D and 3D cell proliferation, colony formation and migration capability, and apoptosis induction. The intratumoral injection of PDMP completely suppressed the growth of OR xenograft models. This study demonstrated that dysregulation of ceramide metabolism is involved in osimertinib-resistance and targeting GCS may be a promising therapeutic strategy for patients progressed to osimertinib.

## Introduction

Lung cancer represents the leading cause of cancer-related death globally^1^, and approximately 85% are Non-Small Cell Lung Cancer (NSCLC).

Among NSCLC, *EGFR* activating mutations occur in about 10–15% and 40–50% of Caucasian and Asian patients, respectively. The most common *EGFR* activating mutations are deletion in exon 19 and missense point mutation in exon 21 (L858R) which lead to the activation of many downstream signaling pathways including MAPK, PI3K/AKT, and STAT involved in the control of cell survival, proliferation, and migration^2^. These mutations confer responsiveness to EGFR tyrosine kinase inhibitors (TKIs) such as gefitinib, erlotinib, afatinib, and dacomitinib. However, despite high sensitivity to first- and second-generation TKIs, acquired resistance develops after a short period of treatment, which is mainly attributed to a secondary point mutation in EGFR catalytic domain, the T790M mutation, occurring in exon 20. Osimertinib is the EGFR mutant selective TKI initially approved for T790M-positive patients and currently used as first-line therapy for advanced *EGFR*-mutated NSCLC patients. Despite high osimertinib efficacy, resistance invariably develops. Acquired resistance to osimertinib are caused by varied mechanisms, including *EGFR*-dependent and -independent resistance mechanisms^3^.

Besides the defined role of genetic mutations and epigenetic changes, onco-metabolism has also been reported to be essential for drug resistance development and maintenance^4–6^.

Glycosphingolipids are bioactive molecules derived from ceramides that play a key role in the regulation of cancer cell signaling contributing to several cellular processes essential for survival^7^. The first step in the biosynthesis of glycosphingolipids is the conversion of ceramide into glucosylceramide (GlcCer) by glucosylceramide synthase (GCS, EC:2.4.1.80). The product of this conversion, a hexosylceramide species (HexCer), is the precursor of the key dihexosylceramide (Hex2Cer) lactosylceramide (LacCer), which has been shown to be involved in cell proliferation^8^, migration and angiogenesis^9^. LacCer also represents the core structure common to trihexosylceramides (Hex3Cer) and thus to nearly all classes of complex glycosphingolipids, including globosides and gangliosides^10^, recently reported to be significant mediators of cancer plasticity^11^*.* Ceramides can also be converted into HexCer metabolites by the action of UGT8 galactosyltransferase enzyme (EC: 2.4.2.62). Galactosylceramide (GalCer) derivatives are next transformed into sulfatides, acidic glycosphingolipids recently reported to concur to cancer malignancy^12^.

GCS has attracted significant attention in oncology, as its overexpression and/or abnormal activity has been reported in various cancers^13^ and found correlated to metabolic derangement in malignant diseases^14^. Moreover, overexpression of GCS has been linked to the occurrence of a resistant phenotype to chemo- or targeted therapy in tumors, ranging from breast^15–17^, leukemia^18,19^, hepatocellular^20^, prostate^21^ and colon^22^ cancers. Furthermore, the inhibition of GCS through the administration of the well-known inhibitor 1-phenyl-2-decanoylamino-3-morpholino-1-propanol (PDMP) has been found to exert significant antitumoral activity^23^ and, in some cases, to revert the drug resistant phenotype in mouse models^24^.

The role of lipid metabolism in lung cancer development and progression has been recently reviewed by our group^25^. Elevated glycosylated ceramide and sphingomyelin species were detected in lung pleural effusions from NSCLC patients^26^, but to date, resistance to clinically approved kinase inhibitors of EGFR has never been associated with the aberrant synthesis of simple or complex glycosphingolipids. In the present study, we investigated whether the insurgence of a resistant phenotype to osimertinib in NSCLC clones can be associated with a significant remodeling in the sphingolipid metabolism compared to drug sensitive lung cancer cells. Moreover, we also examined if in osimertinib-resistant clones, the modulation of the glycosphingolipid levels through the administration of the GCS inhibitor PDMP can exert antiproliferative and proapoptotic effects both in vitro and in vivo.

We found an increment in the levels of glycosylceramides, resulting from a hyperexpression of GCS enzyme in OR models while the inhibition of GCS induced cell cycle arrest, reducing cell proliferation and colony formation, inhibited cell migration and promoted apoptotic cell death. The intratumoral administration of PDMP suppressed the growth of OR xenograft model. Therefore, the use of a GCS inhibitor may represent a promising strategy to treat *EGFR*-mutant NSCLC patients progressed to osimertinib, irrespective of the mechanism of resistance.

## Results

### Characterization of osimertinib sensitive and resistant cell models

**Figure 1:**
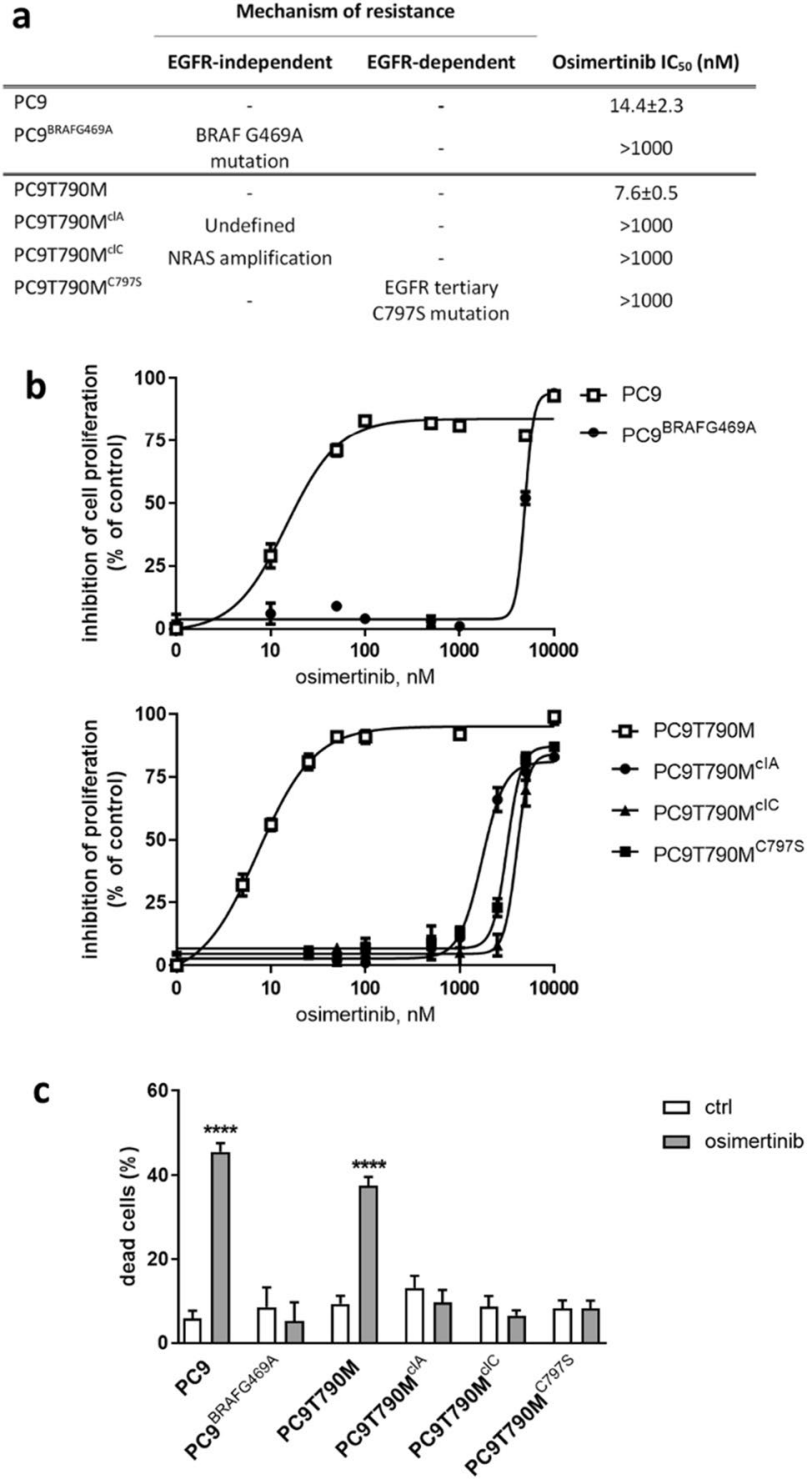
Characteristics of NSCLC osimertinib‐resistant cell lines. (**a**) Mechanisms of resistance to osimertinib (**b**) NSCLC cells were treated with osimertinib (from 10 to 10,000 nM) for 72 h and cell proliferation was assessed by MTT assay. For each cell model the data are expressed as a percentage of inhibition versus the corresponding control cells and are means ± standard deviation (SD) of three independent experiments. (**c**) Cell death was quantified by fluorescence microscopy analysis on Hoechst 33,342 and propidium iodide-stained cells after 72 h of treatment with 30 nM osimertinib for sensitive cells (OS) and 500 nM osimertinib for resistant (OR) cells. Results are means ± SD of three independent experiments. ****p < 0.0001 versus control cells.

This study was performed on a panel of osimertinib-resistant (OR) NSCLC cells originated from EGFR-mutated PC9 and PC9T790M osimertinib-sensitive (OS) cells after long-term treatment with osimertinib. The mechanisms of acquired resistance to osimertinib are reported in Fig. 1a. The OR PC9^BRAFG469A^ cells carry a BRAFG469A mutation, PC9T790M^clA^ have unknown mechanisms of resistance, PC9T790M^clC^ cells carry NRAS amplification and PC9T790M^C797S^ cells have C797S mutation as described in the Materials and Methods section.

OR cells showed an IC_50_ for osimertinib greater than 1000 nM (Fig. 1b) and were resistant to osimertinib-induced apoptosis (Fig. 1c).

### Lipidomic analysis of NSCLC cell lines

An untargeted lipidomic analysis was carried out on cellular extracts obtained from OS or OR cell lines employing a high-resolution mass spectrometry (HRMS) platform (see “Methods” Section). This approach allowed to identify 443 lipid species, common to OS and OR cell lines, belonging to four of the eight categories annotated in the LIPID MAPS database, which included i. fatty acyls (FA; n = 34, corresponding to the 7.7% of total identified lipids); ii. glycerolipids (GL; n = 119, 26.9%); iii. glycerophospholipids (GP; n = 208, 47.0%) and iv. sphingolipids (SP; n = 82, 18.5%).

A partial least squares discriminant analysis (PLS-DA) was performed on normalized ion intensities of identified lipids to search for variables accounting for the different phenotypic response to osimertinib displayed by different clones of NSCLC cells. This statistical approach has been developed to find models able to separate classes of observations based on their X-variables and it has been applied to classify tumour cell populations according to their phenotype^27–29^.

**Figure 2:**
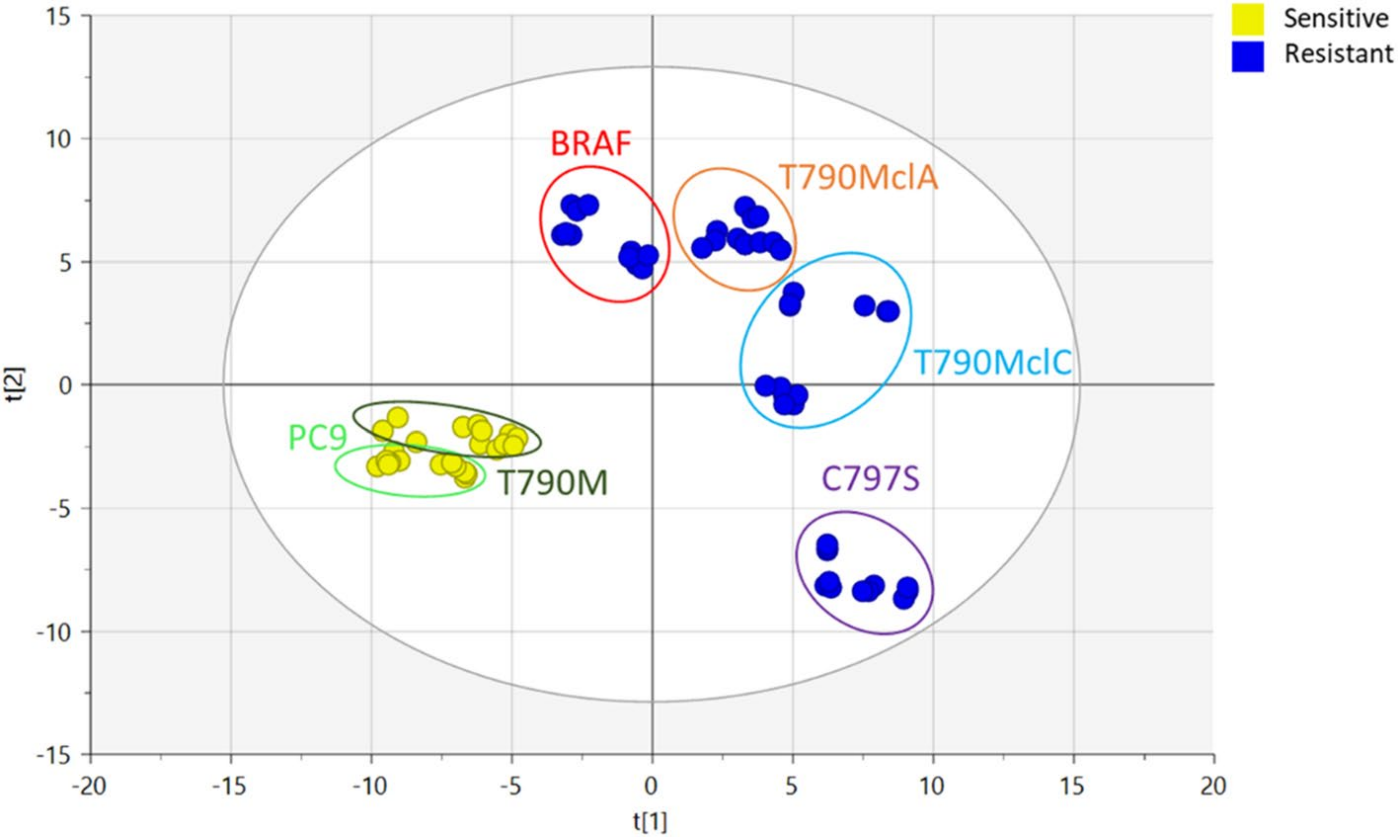
Discriminant analysis (PLS-DA) between OR and OS cell lines: score plot based on identified lipids (n = 443) in cell extracts by UPLC-HRMS on OS (yellow dots) and OR (blue dots) cell lines. For each cell line, four independent samples are represented including technical replicates (n = 3 for each sample). T1 and T2 are orthogonal directions called latent variables derived from the original X matrix maximizing the covariance with the Y response.

The resulting PLS-DA was featured by satisfactory statistical parameters both in description (R2X_cum_ = 0.47; R2Y_cum_ = 0.88) and prediction (Q2_cum_ = 0.87), for the 2-component model. The corresponding score plot (Fig. 2) showed a net separation between OR and OS cells along the first component (*t*_1_)*,* with resistant clones PC9^BRAFG469A^ and PC9T790M^clA^, PC9T790M^clC^, PC9T790M^C797S^ well separated from the corresponding parental cell lines (PC9 and PC9T790M, respectively). The second component (*t*_2_) brought an independent information in the model as it contributed to the separation of PC9T790M^C797S^ clones from all the other OR cell lines.

To identify the variables mostly contributing to the discrimination between the OS and OR groups, we calculated the VIP (variable importance in the projection) parameter for all the X-variables employed in the PLS-DA. The VIP summarizes the importance of the X variables in the response spaces. Lipid signals with a VIP larger than 1 are the most influential for modelling the variation in the response to osimertinib. In our study, we focused our attention on lipids with a VIP value equal or higher than 1.6 (see Table S1). This allowed to restrict the investigation to 30 significant variables, still covering the four categories of lipids initially present in the cellular extracts (i.e., FA, GL, GP and SP). However, a remarkable change in the lipid distribution could be observed after variable screening on VIP basis, with the SP category emerging as the most populated one (n = 13, corresponding to the 43.3% of the most influential lipids), followed by GL (n = 10, 33.3%), FA (n = 4, 13.3%) and GP (n = 3, 10%). Within sphingolipids, the most abundant classes of lipids were neutral glycosphingolipids (such as HexCer and Hex2Cer species, see Table S1), corresponding to the 62% of total SPs with a VIP value equal or higher than 1.6, followed by ceramides (15%), phosphosphingolipids (such as sphingomyelins, 15%), and acidic glycosphingolipids (8%).

### Sphingolipids levels and GCS expression in osimertinib-resistant cells

PLS-DA suggested that the OR phenotype correlated with a reprogramming of the lipid metabolism dominated by significant variations in the levels of glycosphingolipids. We thus performed a set of independent experiments in which we focused our attention on specific glycosphingolipids species (HexCer, Hex2Cer, Hex3Cer) and other relevant sphingolipids (ceramides, sphingomyelins), and we evaluated changes in their levels in each OR cell line in comparison to the relative OS one. Analytical details about identified SP species shared by OR and OS cells including normalized ion intensities (expressed as average values on a log_2_ scale from four independent wells analyzed in triplicate), their fold-changes between OR and OS cell lines and related statistical significance (set at *p* < 0.05 in unpaired two-tailed t-test) are summarized in Supplementary Tables S2–S5 (see Supplementary Materials).

**Figure 3:**
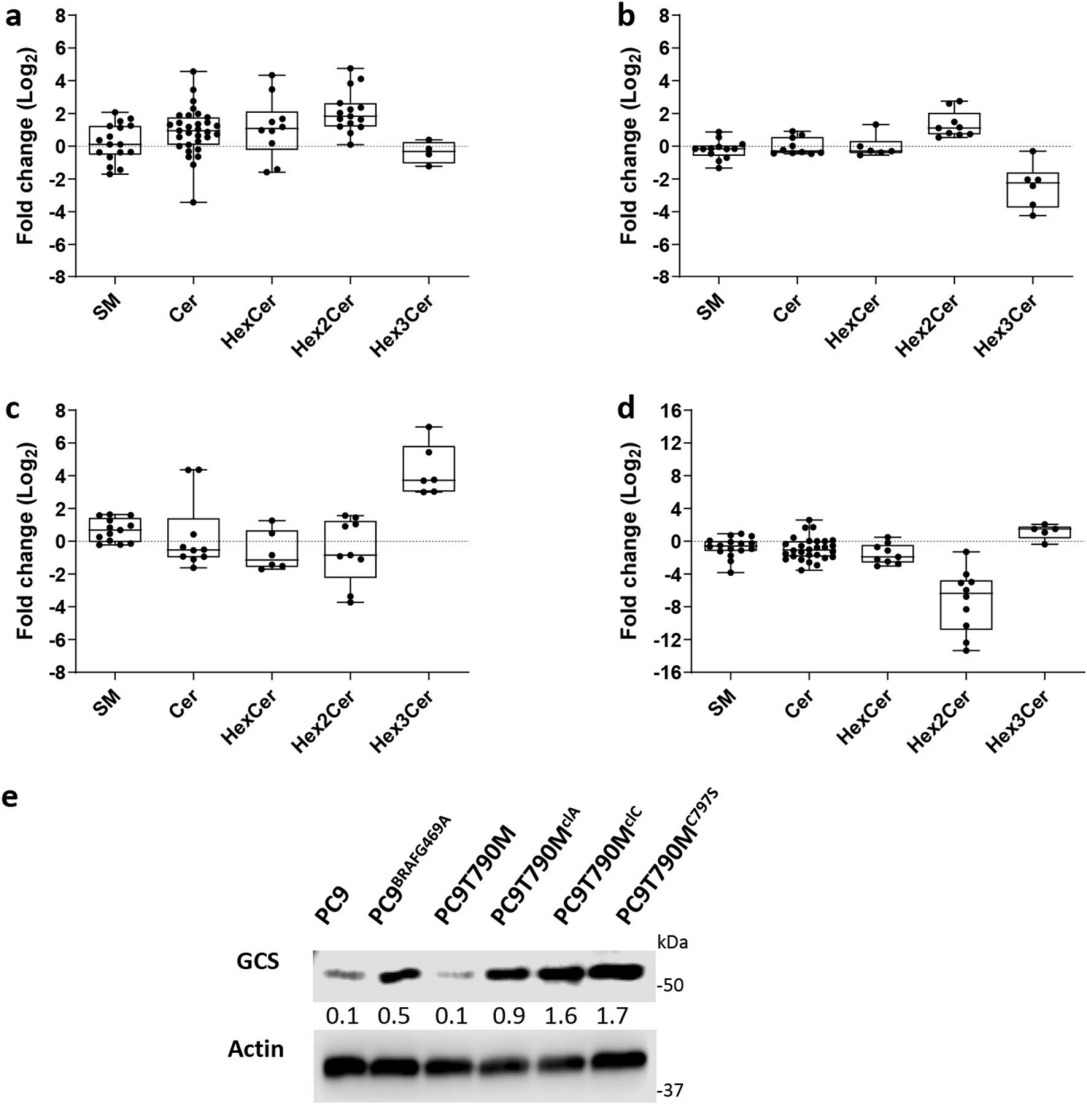
Change of putatively identified sphingolipids and GCS protein level between OR and OS cell lines: Box and whiskers plots for (**a**) PC9^BRAFG469A^ versus PC9; (**b**) PC9T790M^clA^ versus PC9T790M; (**c**) PC9T790M^clC^ versus PC9T790M; (**d**) PC9T790M^C797S^ versus PC9T790M. Sphingolipid species are reported according to their abbreviation in the LIPID MAPS Structure Database: sphingomyelins (SM), ceramides (Cer), hexosylceramides (HexCer), dihexosylceramides (Hex2Cer), trihexosylceramides (Hex3Cer). Fold change is expressed on log_2_ scale. Boxes are determined by the 25th and 75th percentiles and whiskers are determined by minimum and maximum values (**e**) Expression levels of GCS protein and actin were evaluated by Western blotting. Results are representative of three independent experiments. The immunoreactive spots were quantified by densitometric analysis, ratios of GCS/actin were calculated, and values are reported. The blots were cut prior to hybridization with antibodies during blotting. The original blots and replicates are available in Supplementary Fig. S3.

Fold-change values between OR and OS cell lines for key sphingolipids are also reported in Fig. 3. In PC9^BRAFG469A^, we observed a significant increase in the levels of nearly all the identified HexCer (7/10) and Hex2Cer (14/15) lipids, if compared to the parental PC9 cell line (expressed as positive fold change values in Fig. 3a). A similar finding was observed comparing PC9T790M^clA^ with PC9T790M cell lines, although in this case the increment in the glycosphingolipid levels was restricted to the class of Hex2Cer only (9/9, Fig. 3b). Analysis of SP signals in PC9T790M^clC^ and PC9T790M^C797S^ showed a significant increment in the Hex3Cer levels (6/6 for PC9T790M^clC^, 4/5 for PC9T790M^C797S^) in both OR clones (Fig. 3c,d), if compared to the parental PC9T790M cell line. Differently from all the other OR clones, PC9T790M^C797S^ were characterized by a dramatic reduction in the Hex2Cer levels (10/10), a finding that could explain the position of these OR clones in the PLS-DA score plot (Fig. 2).

We thus focused our attention on the ceramide (Cer) class. We observed significant variations in the levels of some ceramides (23/31 in PC9^BRAFG469A^ cells; 1/10 in PC9T790M^clA^; 7/10 in PC9T790M^clC^ and 22/28 in PC9T790M^C797S^, see Fig. 3A–D). However, differently from what observed for glycosylceramides, we could not detect a clear class trend able to discriminate OR from OS clones. A similar finding was also observed for sphingomyelins (SM).

We aimed to assess the reproducibility of the analytical approach by performing a new experiment in which SP signals in PC9T790M^clC^ clone were compared to that of PC9T790M cell line, similarly to what previously reported and summarized in Fig. 3c. A similar trend in the relative SP levels was observed between the two sets of experiments (Fig. S1).

The alteration in the pool of identified glycosylceramides in OR cells, accompanied by an elevation of the levels of dihexosylceramides (Hex2Cer) in PC9^BRAF469A^ and PC9T790M^clA^ and trihexosylceramides (Hex3Cer) in PC9T790M^clC^ and PC9T790M^C797S^ clones prompted us to investigate whether osimertinib resistance could be associated with an alteration in the expression of GCS, the enzyme catalyzing the first key step of the conversion of ceramide into HexCer. As shown in Fig. 3e, increased GCS level expression, as evaluated by Western blot analysis, was found in all the investigated OR clones, if compared to PC9 or PC9T790M OS parental cells.

### Effect of PDMP on glycosylated ceramides and other sphingolipids

**Figure 4:**
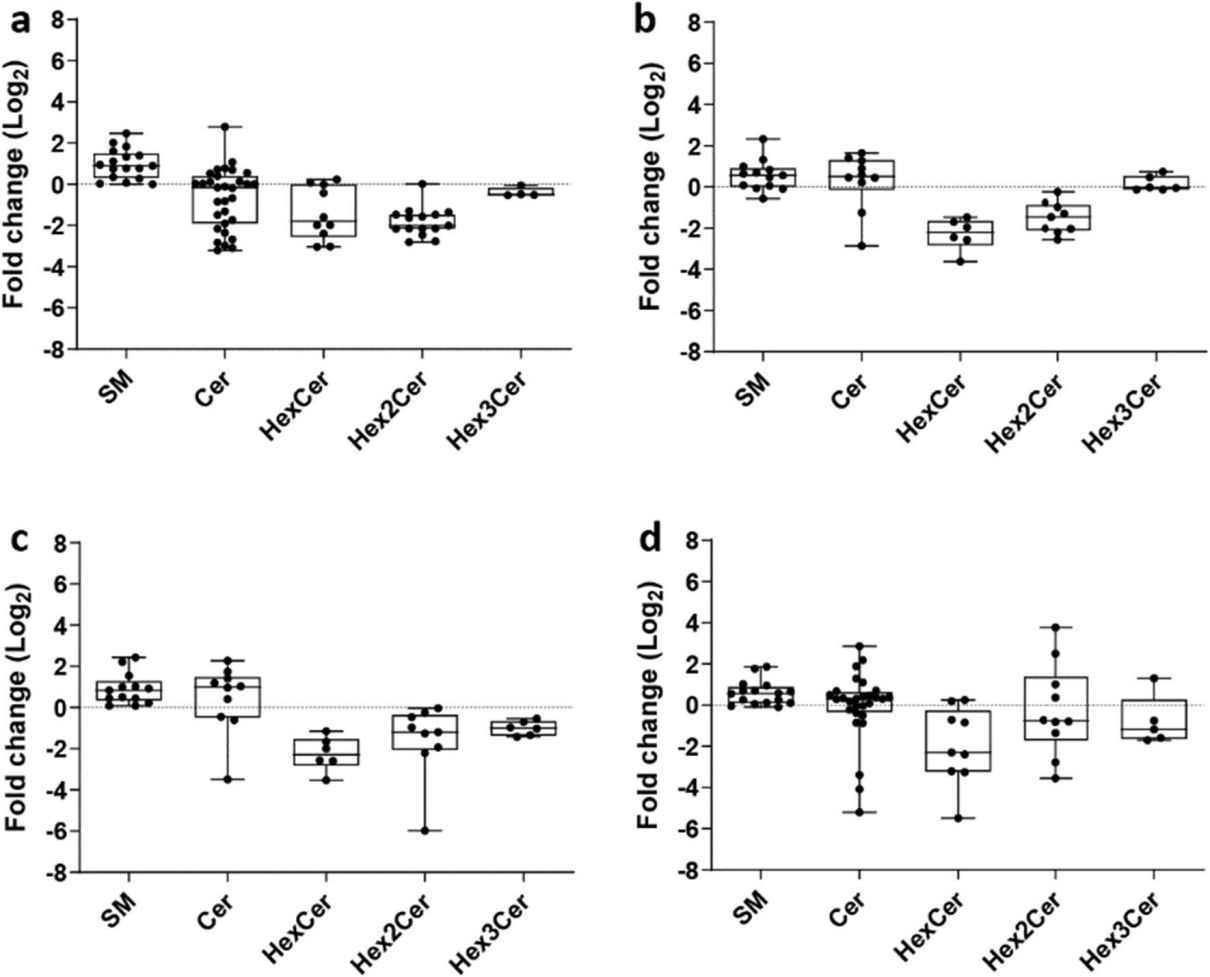
Change of putatively identified sphingolipids between OR cell lines treated or not with 20 µM PDMP: Box and whiskers plots for (**a**) PC9^BRAFG469A^ + PDMP versus PC9^BRAFG469A^; (**b**) PC9T790M^clA^ + PDMP versus PC9T790M^clA^; (**c**) PC9T790M^clC^ + PDMP versus PC9T790M^clC^; (**d**) PC9T790M^C797S^ + PDMP versus PC9T790M^C797S^. Fold change is expressed on log_2_ scale. Boxes are determined by the 25th and 75th percentiles and whiskers are determined by minimum and maximum values. Sphingolipid species are reported according to their abbreviation in the LIPID MAPS Structure Database: sphingomyelins (SM), ceramides (Cer), hexosylceramides (HexCer), dihexosylceramides (Hex2Cer), trihexosylceramides (Hex3Cer).

We thus evaluated whether a treatment with a GCS inhibitor could affect the levels of specific classes of sphingolipids in OR cell lines. We thus designed an experiment in which PC9^BRAFG469A^, PC9T790M^clA^, PC9T790M^clC^, PC9T790M^C797S^ cell lines were treated with 20 µM PDMP for 24 h and underwent a lipid extraction procedure. Following UPLC-HRMS analysis on lipid extracts, fold changes in normalized ion intensities for glycosylated ceramides (HexCer, Hex2Cer and Hex3Cer), ceramides and sphingomyelins in OR clones treated with PDMP or vehicle were reported in Fig. 4. Also, for this set of experiments, analytical and statistical details on identified SP species are summarized in Supplementary Tables S6–S9 (see Supplementary materials).

PDMP significantly modified the levels of glycosylated ceramides expressed by OR clones, as shown by the fold-change box plots reported in Fig. 4. The treatment induced a significant drop in both HexCer and Hex2Cer levels in all the OR clones, except for few Hex2Cer expressed by PC9T790M^C797S^ cell line. PDMP was also able to reduce Hex3Cer levels, although this phenotypic change was restricted to PC9T790M^clC^ and PC9T790M^C797S^ OR clones. Treatment with PDMP somehow affected the expression of the SM species, with identified sphingomyelins significantly upregulated or remaining at the same level of the untreated cells, indicating the presence of a common response in all the OR clones. Ceramide levels were also influenced by the treatment with PDMP although a common trend to all OR clones could not be detected.

### Effect of GCS inhibition on 2-D and 3-D cell growth, cell survival and cell migration

**Figure 5:**
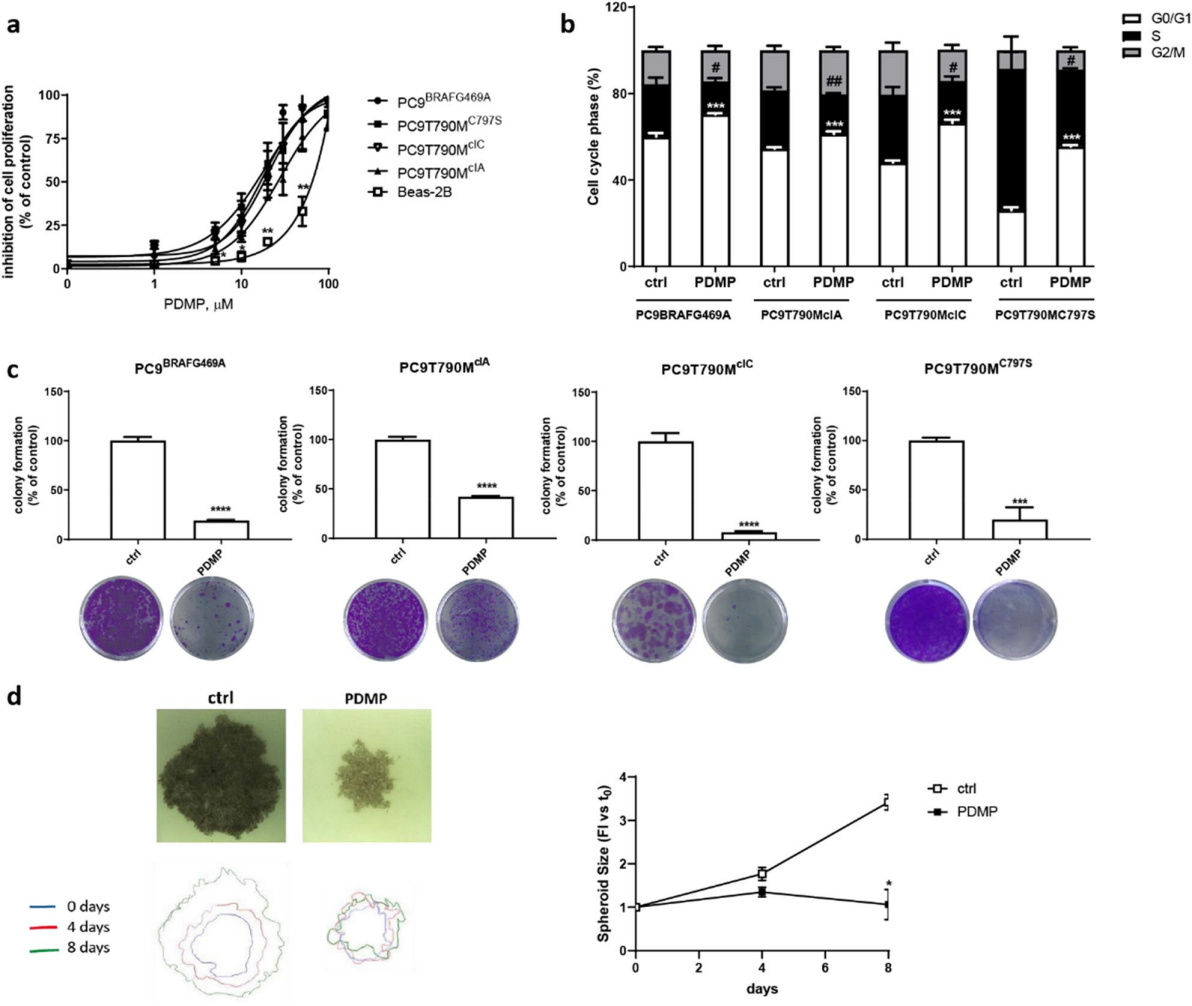
Effects of PDMP on cell proliferation, colony formation and 3-D growth: (**a**) OR cell lines and BEAS-2B cells were treated with increasing concentrations of PDMP for 72 h. Cell proliferation was assessed by MTT assay; for each cell model, the data are expressed as a percentage of inhibition versus the corresponding untreated control cells and are means ± SD of three independent experiments. *p < 0.05; ** *p* < 0.01; versus OR cells. (**b**) The indicated cell lines were treated with 20 µM PDMP for 24 h. Then, the cells were stained with PI and their distribution in cell cycle phases was determined by flow cytometry. Results are means ± SD of three independent experiments. ***p < 0.001 versus G_0_/G_1_ phase; #*p* < 0.05; ## < 0.01 versus S phase of corresponding control cells. (**c**) The indicated OR cells were treated with 20 µM PDMP and after 10 days colony formation was assessed as described in the Materials and Methods section. Representative images of CV staining of colonies are shown. Data are the means ± SD of at least three independent experiments. (**d**) The growth of spheroids from PC9^BRAFG469A^ cells was analyzed after 4 and 8 days of treatment with 20 µM PDMP. Data are expressed as fold increase (FI) versus T_0_. Representative images of spheroids after 8 days of culture are shown. Data are representative of two independent experiments. *p < 0.05; ***p < 0.001; ****p < 0.0001 versus control cells.

We then tested the responsiveness to GCS inhibitor PDMP in terms of cell proliferation inhibition and cell cycle arrest. All OR cell clones, regardless of the mechanisms of resistance, showed sensitivity to PDMP, with IC_50_ values ranging from 15 to 25 µM (Fig. 5a). Interestingly, normal bronchial BEAS-2B cells were significantly less sensitive to PDMP treatment than OR tumor cells (IC_50_ 60 µM). We also tested another GCS inhibitor currently in clinical use, eliglustat, which inhibited cell proliferation although to a lesser extent than PDMP, with IC_50_ ranging from 25 to 50 µM (Fig. S2).

As shown in Fig. 5b, PDMP induced a significant blockade of the progression from G_1_ to S phase of the cell cycle increasing the fraction of cells in the G_0_/G_1_ phase and decreasing the fraction in the S-G_2_/M phase in all OR cells. The effect of PDMP was also evaluated on the ability of cells to form colonies. By the colony formation assay, we confirmed the efficacy of PDMP in reducing the number of colonies after 10 days of exposure to the drug in all OR cell models (Fig. 5c).

Furthermore, we also evaluated the anti-proliferative activity of PDMP in three-dimensional (3D) systems. We generated tumor spheroids from PC9^BRAFG469A^ and as shown in Fig. 5d, we demonstrated that, PDMP significantly inhibited the growth of tumor spheroids after 8 days of culture.

**Figure 6:**
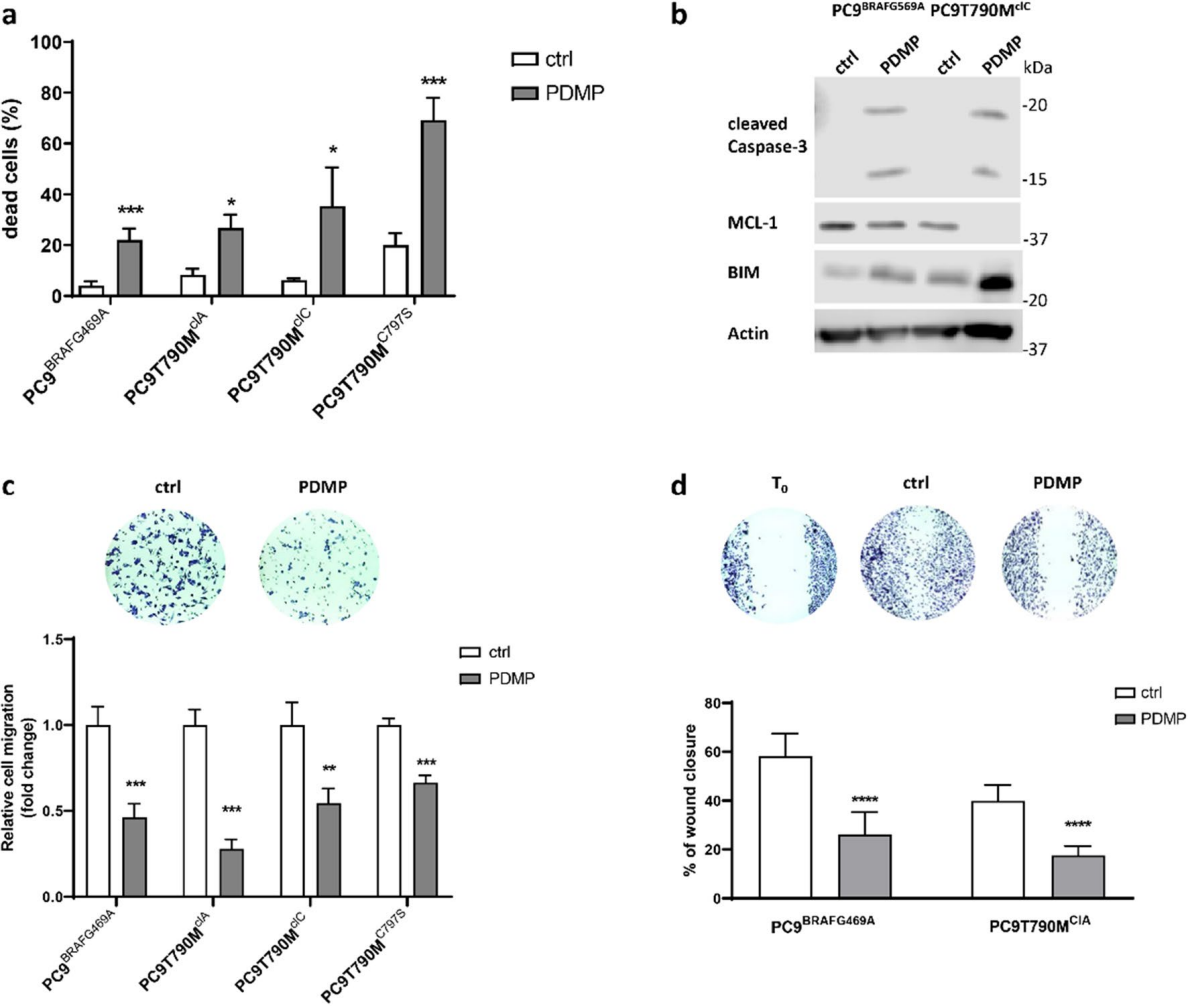
Effects of PDMP on cell death and cell migration: (**a**) OR cells were treated with 20 µM PDMP for 72 h and cell death was quantified by fluorescence microscopy analysis on Hoechst 33,342 and PI-stained cells. Data are representative of at least two independent experiments. (**b**) The indicated OR cells were treated with 20 µM PDMP 72 h, then lysed and western blot analysis was performed to detect the cleaved caspase 3, MCL-1, BIM and actin proteins. Results are representative of two independent experiments. The blots were cut prior to hybridization with antibodies during blotting. The original blots and replicates are available in Supplementary Fig. S4. (**c**) Cells were seeded in 8 µm pore transwell and after 16 h of treatment with 20 µM PDMP, cells that have migrated through the membranes were counted under a Phase contrast microscope. Results are expressed as fold change compared to untreated cells. Representative images are shown. (**d**) The indicated OR cells were treated with 20 µM PDMP for 24 h, then a wound healing was performed in each well as described in Material and Methods section. Representative images were taken at T_0_ and after 24 h of treatment. Percent closure was calculated as wound area 24 h/wound area T_0_ × 100. *p < 0.05; **p < 0.01; ***p < 0.001; ****p < 0.0001 versus control cells.

The ceramide pathway has been reported to play an important role in mediating cell death by apoptosis. Considering that OR cells were highly resistant to osimertinib-induced apoptosis (see Fig. 1c), we evaluated the role of PDMP in inducing cell death. As shown in Fig. 6a, the percentage of dead cells significantly increased after exposure to PDMP for 72 h in all the OR cell lines with values ranging from 22% for PC9^BRAFG469A^ to 70% for PC9T790M^C797S^ cells. Cell death occurred through apoptosis, as indicated by the activation of caspase-3 (Fig. 6b). In addition, the anti-apoptotic protein Mcl-1 was down-regulated, and the pro-apoptotic protein BIM was upregulated.

Finally, considering the role of GCS activity in tumor metastasis^30^, we evaluated the effect of the treatment with PDMP on cell migration. The OR cells in the presence of PDMP showed a significant decrease in the number of migrating cells through the PET membrane in Boyden chambers (Fig. 6c). This result was confirmed by wound healing assay. Indeed, PDMP-treated cells spread into the wound area less efficiently than control cells, with wound closure percentages of 26% versus 58% in PC9^BRAFG469A^ cells and 17% versus 40% in PC9T790M^clA^ cells (Fig. 6d).

### In vivo assessments of PDMP efficacy

Before investigating the antitumoral activity of PDMP in a xenograft mice model of NSCLC resistant to osimertinib, we measured plasmatic concentration of this compound in a cohort of healthy nude mice (n = 3 per time point) after the intraperitoneal (i.p.) administration of a 100 mg/kg dose. A 3 mg/kg dose of osimertinib was also administered to the same cohort by oral gavage.

**Figure 7:**
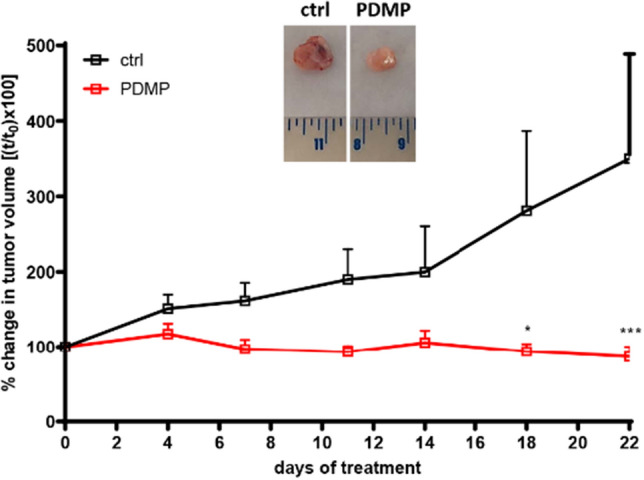
Effect of PDMP treatment on tumor growth: 5 × 10^6^ PC9^BRAFG469A^ cells were subcutaneously injected in the flank of BALB/c-Nude female mice and when tumor volume reached an average size of 150 mm^3^, the animals were randomized into two groups: control (n = 5), PDMP (n = 7) treated mice.PDMP (5 mg/kg dose) was intratumorally administered every day. All mice were treated daily with osimertinib (3 mg/kg; oral gavage). Tumor xenografts were measured twice a week and data are expressed as percent change in tumor volume ± SEM. (*p < 0.05; ***p < 0.001 versus Ctrl; two-way ANOVA followed by Bonferroni’s post-test). Representative images of dissected xenograft tumors are shown.

One hour after i.p. injection, the plasma concentration of PDMP overcame the micromolar range (mean ± SD = 5.5 ± 1.0 µM), while after 3 h PDMP levels dropped far below this limit (0.1 ± 0.04 µM), suggesting a rapid clearance from plasma. This prompted us, also supported by literature data^31^ to design an in vivo experiment in which PDMP was delivered to the mice by intratumoral administration. PC9^BRAFG469A^ were subcutaneously injected into BALB/c nude female mice, and when tumors reached an average volume of 150 mm^3^, animals were randomly assigned into two groups: control (n = 5) and PDMP-treated mice (n = 7). All mice were treated daily with osimertinib (3 mg/Kg; oral gavage). The intratumoral injection of a daily dose of 5 mg/kg of PDMP completely suppressed the tumor growth (Fig. 7) and a statistical significance was reached after 18 days of treatment.

## Discussion

For advanced NSCLC patients carrying *EGFR* mutations, osimertinib represents the standard of care as first-line treatment. Despite its efficacy, after about 18 months resistance is expected and several heterogeneous mechanisms have been described^3^. It has been demonstrated that *EGFR* mutations can drive metabolic alterations^32,33^ and metabolic reprogramming may exert an important role in EGFR-TKIs resistance^34^, as recently reviewed by our group for lipid metabolism^25^.

In our work here, an untargeted lipidomic analysis showed a significant alteration of the lipidome in OR cell lines when compared to the OS ones with neutral glycosphingolipids, particularly HexCer, Hex2Cer and Hex3Cer derivatives, significantly upregulated in OR cells.

Alterations in the lipidome of EGFR-TKI resistant cells (compared to sensitive ones) have been already described. A comparison between PC9 and PC9/GR (gefitinib resistant cells) has revealed a significant difference in lipid groups with modification in the proportion of saturated and unsaturated phospholipids^35^. The activation of the lipogenic pathway by the Sterol Regulatory Element Binding Protein-1 (SREBP-1) was found to contribute to gefitinib-acquired resistance and targeting SREBP-1-induced lipogenesis was able to overcome such resistance^35^. The involvement of SREBP-1/ACC/FASN axis in osimertinib resistance has been demonstrated by Chen et al.^36^. In OS cells, osimertinib promoted degradation of SREBP-1, significantly decreasing the levels of FASN and ACC, and reducing the content of lipid droplets (LDs). By contrast, OR cells showed elevated levels of SREBP-1, FASN, and ACC and sustained lipid metabolism.

Gefitinib-resistant NSCLC clones showed a higher content of LDs than the parental gefitinib-sensitive PC9 and HCC827 cells, suggesting a role of LD accumulation in gefitinib-resistance. Interestingly, the inhibition of SCD1, by reducing LD formation reverted resistance, inducing apoptosis in vitro and inhibiting tumor growth in vivo^37^.

Alterations in sphingolipid metabolism playing a major role in drug resistance may include changes in ceramide metabolism-associated enzymes and decreased levels of ceramides^13^. In lung cancer, metabolic alterations in sphingolipids were found to affect cancer progression, development, and drug resistance, but until now no data in the literature associate resistance to EGFR-TKIs with aberrant ceramide glycosylation. A study conducted by Zhang in 2014 found that the expression of GCS was increased in 64.4% of NSCLC specimens compared to the non-cancerous specimens and was associated with poor prognosis, lymph node metastasis and resistance to chemotherapy^38^. Expression of GCS has been found to be associated with multi-drug resistance in many tumours including NSCLC^7^ and in our OR models increased levels of GCS were observed in comparison with parental OS cell lines.

GCS has an important role in the regulation of resistance against cisplatin in NSCLC and mesothelioma cells. The synthesis of globotriaosylceramides (Gb3s) was found to be increased in cisplatin-resistant H1299 NSCLC and the use of GCS inhibitors sensitized this cell line to cisplatin^39^. Chiu et al. demonstrated that vinorelbine-resistant cell lung adenocarcinoma has overexpressed GCS and, after treatment with PDMP or GCS silencing, an accumulation of ceramide and decreased glucosylceramides levels were accompanied by apoptosis with a reduction in anti-apoptotic Bcl-xL protein expression^40^.

The treatment of our OR cell lines with PDMP led to the arrest in the G_0_/G_1_ phase of the cell cycle with inhibition of cell proliferation and colony formation capability. In addition, PDMP treatment induced cell death by apoptosis, as suggested by microscopy analysis and confirmed by cleavage of caspases 3, accumulation of late apoptotic protein BIM, and downregulation of anti-apoptotic protein MCL-1.

The mechanisms by which inhibition of glycosylceramide synthesis inhibits cell proliferation and induces cell death have been generally associated to ceramide accumulation leading to activation of antiproliferative and pro-apoptotic pathways^41^. By contrast, our results indicated decreased levels of glycosylceramides during PDMP treatment, but no clear changes in the levels of ceramides. The mechanisms behind the activation of apoptosis upon inhibition of GCS in resistant cells need to be better clarified, however, it could be attributed to the role of glycosylceramides per se in cell death and survival. Interestingly, in breast cancer it has been demonstrated that the increase of galactosylceramide by itself, regardless of ceramide levels, was a pro-survival mechanism^42^ and galactosyl ceramide conversion to sulfatide was directly associated with highly increased sensitivity to pro-apoptotic agents^43^. Moreover, GCS overexpression altered the composition of glycosphingolipid-enriched microdomains (GEMs) with accumulation of GlcCer and Gb3 and activation of the pro-survival AKT and ERK1/2 pathways^16^.

To further investigate the effects of inhibition of the accumulation of glycosylceramides in resistant cells, we evaluated spheroid formation and cell migration upon PDMP treatment. We observed a significant inhibition of spheroid formation, besides a significant inhibition of cellular migration and wound healing providing a possible benefit of inhibition of the synthesis of glycosylceramides in reducing invasiveness. GCS overexpression was detected in breast cancers with metastasis, but not in benign fibroadenoma or primary tumors^17^ and PDMP treatments significantly reduced wound healing of TP53 mutant-doxorubicin resistant colon cancer cells^44^. Our in vivo results confirmed the efficacy of PDMP in suppressing tumor growth in OR xenograft mouse models.

In conclusion, our results suggest that an increased production of glycosylceramides contributes to the survival of clones resistant to osimertinib through the inhibition of apoptotic cell death, and the use of a GCS inhibitor may represent a promising strategy to treat *EGFR*-mutant NSCLC patients who have progressed to osimertinib, regardless of the mechanism of resistance. Additionally, GCS inhibitors, such as eliglustat, are a clinically available treatment option for Gaucher disease, a rare inherited metabolic disorder in which deficiency of the enzyme glucocerebrosidase results in lysosomal accumulation of glucocerebrosides^45^. The favorable safety profile of eliglustat could suggest its use also for the treatment of NSCLC patients progressed to osimertinib treatment^46^.

## Methods

### Cell lines and culture

The NSCLC cell line PC9 was kindly provided by Dr P. Jänne (Dana-Farber Cancer Institute, Boston MA, USA). PC9T790M cell clone was generated in our lab by exposing PC9 to increasing concentrations of gefitinib^47^.

The resistant clones showing different mechanisms of acquired resistance to osimertinib (Fig. 1a) were generated as previously described^48–51^. The osimertinib-sensitive PC9 and PC9T790M cells were exposed to increasing concentration of osimertinib for approximately 9–12 months, and then the resistant clones (able to grow in the presence of 500 nM osimertinib) were selected and characterized for the resistance mechanisms. The osimertinib-resistant clone PC9T790M^C797S^ was kindly provided by Dr. Mancini (Institut de Recherche en Cancérologie de Montpellier (IRCM)-Université de Montpellier, France). Cells were cultured in RPMI-1640 (Dominique Dutscher SAS, Bernolshim, France) medium supplemented with 10% fetal bovine serum (Invitrogen, Carlsbad, CA, USA) and maintained under standard cell culture conditions at 37 °C in a water-saturated atmosphere of 5% CO_2_ in air. Resistant cells were routinely cultured in the presence of 500 nM osimertinib to maintain a selection pressure during in vitro propagation. All experiments were performed in the presence of 500 nM osimertinib.

### Reagents and chemicals

Ultra-pure water was obtained by a Milli-Q plus system from Millipore (Bedford, MA, USA). LC–MS grade acetonitrile (MeCN), methanol (MeOH), isopropanol (IPA) and ammonium acetate were purchased from Scharlab (Barcelona, Spain), while chloroform and Phosphate Buffer Saline (PBS) tabs were purchased from Sigma-Aldrich (St Louis, MO, USA). Major Mix IMS/TOF calibration kit and Leukin-Enkephalin (Leu-Enk) solution from Waters (Manchester, UK) were employed for external and internal calibration of the mass spectrometer. Lipidomix® kit was purchased from AVANTI Polar (Alabaster, AL, USA). Osimertinib was provided by AstraZeneca (Milan, Italy). PDMP, namely, DL-threo PDMP (a mixture of D-threo-(1R,2R)-PDMP and L-threo-(1S,2S)-PDMP isomers) was purchased from Cayman Chemical (Ann Arbor, MI, USA). Stock solutions were prepared at a convenient concentration by dissolving the required amount of the titled compound in DMSO (Sigma Aldrich), aliquoted to reduce the freeze–thaw cycles, and diluted in fresh medium before use. Final DMSO concentration in medium never exceeded 0.1% (v/v).

### Analysis of cell proliferation, cell death and cell cycle

Proliferation and viability of cells were evaluated by tetrazolium dye [3-(4,5-dimethylthiazol-2-yl)-2,5-diphenyltetrazolium bromide] (MTT) and Crystal violet (CV) assays as previously described^50^. Cell death, assessed by fluorescence microscopy analysis after dual staining with Hoechst 33,342 and propidium iodide (PI), and distribution of the cells in the cell cycle, determined by PI staining and analyzed by the CytoFLEX Flow Cytometer (Beckman Coulter Life Sciences, Indianapolis, IN, USA) were described elsewhere^52^.

### Spheroid generation

To generate 3D spheroids, U-shaped 96-well plates were covered with 80 µL of warm 1.5% agarose in PBS. After 30 min 1.5 × 10^3^ cells were added in 80 µL of medium and after 24 h drugs were added to the medium. Pictures of spheroids were taken every 96 h using Nikon Eclipse E400 Microscope with digital Net camera. Spheroids sizes were calculated using ImageJ software (NIH, MD, USA).

### Colony formation assay

Cells were seeded in 6-well plates at a density of 10^3^ cells per well. After 10 days of treatment with drugs, colonies were fixed with ice-cold methanol and stained with 0.1% CV (Sigma Aldrich). The unbound dye was removed by washing with water. The bound CV was solubilized with 0.2% TritonX-100 in PBS and the absorbance of the solution was measured at a wavelength of 570 nm^50^.

### Cell migration and wound healing assays

The migration assay was carried out using 6.5 mm Transwell^®^ with 8.0 µm Pore Polycarbonate Membrane Insert (Corning, NY, USA) as previously described^53^. Briefly, 10^5^ cells were loaded in the upper wells. After 16 h, cells that have migrated through the PET membranes were fixed with 100% methanol, stained with hematoxylin and counted under a Phase contrast microscope.

Wound-healing assay was performed with the CytoSelect 24-well Wound Healing Assay Kit (Biolabs, San Diego, CA, USA) according to the manufacturer’s instruction as described previously^53^. Images of wound healing were captured by microscope equipped with digital camera (magnification 40x) at zero time point and after 24 h. Cell migration was quantified by measuring the migration distances using ImageJ software: for each picture, the mean of 5–7 different distance points throughout the entire picture was calculated by the software. Percent closure was calculated as wound area 24 h/wound area zero time point × 100.

### Western blot analysis

Western blot was conducted as described previously^50^. Antibodies against cleaved Caspase-3 (9661; 1:1000), BIM (2933; 1:1000), MCL-1 (94296; 1:1000), actin (3700; 1:1000), and HRP-conjugated secondary antibodies were purchased from Cell Signaling Technology (Beverly, MA, USA), GCS (12869-1-AP; 1:1000) was from Proteintech (Rosemont, IL, USA); the chemiluminescence system (ImmobilionTM Western Chemiluminescent HRP Substrate) was from Millipore. Reagents for electrophoresis and blotting analysis were from BIO-RAD (Hercules, CA, USA). The chemiluminescent signal was acquired by C-DiGit^®^ Blot Scanner and the spots were quantified by Image Studio™ Software, LI-COR Biotechnology (Lincoln, NE). The blots were cut prior to hybridization with antibodies during blotting. The original blots and replicates are available in supplementary information.

### Lipidomic analysis

#### Extraction of cell lipidome

Each cell line was harvested in four independent wells. Cell pellets were extracted from each well employing a modified Folch method, similar to that reported by Eirikkson et al.^54^. Briefly, cells were suspended in growth medium and transferred into glass tubes, washed with ice-cold PBS and centrifuged (3000 g, 3 min, 4 °C). Washed cell pellets were thus extracted twice by a mixture of chloroform:MeOH:water (1:1:1; v/v/v). Chloroform bottom layers from both extractions were pooled, dried under nitrogen stream and reconstituted into a fixed volume of MeCN:IPA:water (1:2:1; v/v/v) before Ultra Performance Liquid Chromatography-High Resolution Mass Spectrometry (UPLC-HRMS) analysis.

#### UPLC-HRMS data acquisition

Cell extracts were analyzed employing an Acquity UPLC system (Waters, Milford, USA), coupled to a Vion IMS-QTof mass spectrometer (Waters, Manchester, UK) equipped with electrospray ionization (ESI) probe in high definition HDMS^E^ acquisition mode. An Acquity HSS T3 UPLC column (2.1 × 100 mm; 1.8 μm particle size; Waters, Milford, USA), thermostated at 55 °C, was used for gradient separation. Mobile phases were A: MeCN:water (40:60 v/v) and B: IPA:water:MeCN (90:5:5 v/v), both containing 5 mM ammonium acetate and 0.1% v/v acetic acid. A linear gradient was employed from 40 to 100% B during the first 14 min, followed by a column clean up at 100% B for 3 min and reconditioning at the initial conditions for 3 min. The total run time was 20 min at a flow rate of 0.4 mL/min. The autosampler temperature was kept at 4.0 °C and injected volume was 5 µL. The capillary voltage was set to 2.5 kV, cone voltage to 40 V. Source temperature was 120 °C; desolvation gas (N_2_) temperature was set at 500 °C at a flow rate of 1000 L/h; source gas (N_2_) flow rate was 50 L/h. Mass range was 50–1200 Dalton, with a scan time of 0.2 s and an interscan delay of 0.02 s. Reference lock mass calibration (50 ng/mL Leucine enkephalin) was used to ensure mass accuracy throughout the analysis. 10 µL of each sample were combined to prepare quality control (QC) samples, which were run before the analytical batch and every six samples during the analysis. All the samples were randomized prior to analysis. Acquisition occurred in electrospray positive (ESI^+^) and the software UNIFI v.1.8.2 (Waters) was used for both system control and data acquisition. Each cell extract was analyzed in three technical replicates.

#### Lipidomic data pre-processing and filtering

Acquired lipidomic data were processed by the software Progenesis QI v. 2.3 (Nonlinear Dynamics, Newcastle, UK). UNIFI *.uep* data files were uploaded and UPLC-HRMS runs were aligned using the pooled QC samples as alignment references. The retention time window for peak picking algorithm was set between 1.5 and 17.0 min, minimum peak width to 0.025 min and sensitivity of the peak picking algorithm was set to 3. For normalization, the default setting in Progenesis QI “normalize to all compounds” was applied. This normalizes all the peak abundances in a sample by the same normalization factor, which is calculated on the basis of peaks present in both the reference QC sample and the sample itself. Other parameters were set to default. With these settings, Progenesis QI returned 4038 ion features characterized by a combination of retention time and *m/z*. Only those ion features with a coefficient of variation (CV%) ≤ 25% in the QC samples and *m/z* ≥ 350 Da were selected; this resulted in 2205 ion features.

#### Lipid identification and annotation

Individual lipids were identified by extensive search into publicly available databases (i.e., Human Metabolome database, HMDB^55^; LIPID MAPS^56^) according to Progenesis QI compound identification algorithm. The UPLC-HRMS analytical method allowed the detection of lipid species belonging to four of the eight categories annotated in the LIPID MAPS database^57^; they included fatty acyls (FA), glycerolipids (GL), glycerophospholipids (GP) and sphingolipids (SP). Lipids were annotated as follows: ceramides (Cer), sphingomyelins (SM), hexosylceramides (HexCer), dihexosylceramides (Hex2Cer), trihexosylceramides (Hex3Cer), fatty esters of hydroxy fatty acids (FAHFA), glycerophosphocholines (PC), glycerophosphoinositols (PI), glycerophosphoglycerols (PG), triacylglycerols (TG) and wax monoesters (WE). Individual lipids were annotated following the LIPID MAPS classification system at the species level indicating: (i) lipid species; (ii) total carbon number in fatty acyl chains; (iii) total number of double bonds. For each lipid, *m/z* ratio, adducts formed, elemental formula, mass error (in ppm), isotopic similarity versus calculated isotopic pattern and mass fragmentation were obtained and IDs associated with an overall Progenesis QI score ≥ 40 (n = 443) were filtered and submitted to multivariate data analysis.

#### Multivariate data analysis on identified lipid signals

Partial least squares discriminant analysis (PLS-DA) was performed using SIMCA v. 17 software (Sartorius Stedim Biotech, Sweden). The analysis included 443 identified lipid signals. Data were log_10_-transformed, centered and pareto scaled prior to modelling. A Variable Importance in Projection (VIP) > 1.6 was employed as a cut-off value to select the most discriminant lipid signals.

#### Univariate data analysis on selected sphingolipid species

Individual lipid signals belonging to the sphingolipid species of sphingomyelins (SM), ceramides (Cer), hexosylceramides (HexCer), dihexosylceramides (Hex2Cer), trihexosylceramide (Hex3Cer) were submitted to univariate data analysis. Average log_2_ intensity values together with their SD were reported for each lipid signal in each cell line (n = 4 per cell line). Unpaired two-tailed Student’s t-test was employed to search for statistically significant differences between osimertinib-sensitive (OS) and osimertinib-resistant (OR) clones and to evaluate the effect of treatment with the GCS inhibitor PDMP. Obtained *p*-values are reported for each comparison in the Supplementary Materials.

### PDMP quantification in mouse plasma

The GCS inhibitor PDMP was extracted from plasma by protein precipitation adding a double volume of ice-cold acetonitrile containing 100 nM deuterated palmitoylethanolamide as internal standard (PEA-d_4_). Samples were analyzed by a Thermo Quantum Access Max triple quadrupole mass spectrometer (Thermo Fisher Scientific, Waltham, MA, USA) equipped with a H-ESI interface acquiring in electrospray positive (ESI^+^) and in multiple reaction monitoring (MRM) mode. HPLC separation occurred by a linear gradient employing a Waters X Select T3 column (2.1 X 100 mm; 3.5 µm particle size; Waters, USA). Eluent A: acetonitrile + 0.1% v/v HCOOH; eluent B: water + 0.1% HCOOH). HPLC Gradient was as follows: t = 0 min: 5%A; t = 1 min: 5%A; t = 5 min: 100%A; t = 8.5 min: 100%A; t = 9.5 min: 5%A with a 2.5-min reconditioning time. Total run time: 12 min. Flow rate: 0.22 mL/min; injection volume: 10 µL. Instrumental parameters were set as follows: ion source voltage: 4000 V; Capillary temperature: 270 °C; sheath gas (N_2_): 35 psi; auxiliary gas (N_2_): 15 psi; collision gas (Ar) pressure: 1.5 mtorr. Tube lens voltages (TL) and collision energies (CE) for each parent-product ion transition were optimized by Flow Injection Analysis (FIA). The following transitions were employed: PDMP: *m/z* = 391.3 [M + H]^+^ → *m/z* = 304.2, 286.2, 100.3 (TL: 89 V; CE: 18, 21, 28 V, respectively); IS: *m/z* = 304.3 [M + H]^+^ → *m/z* = 66.3 (TL: 72 V; CE: 15 V). Calibration curves for PDMP were built by spiking standard dilutions of PDMP stock solution in DMSO into pooled control plasma to span a concentration range between 1000 and 10 nM. Coefficient of determination (r^2^) was > 0.99 for all calibration curves.

### In vivo experiments

A total of 5 × 10^6^ PC9^BRAFG469A^ cells were suspended in 200 μL of Matrigel (BD Biosciences, Erembodegem, Belgium) and PBS (1:1) and were subcutaneously injected in the flank of BALB/c-Nude female mice (Envigo, Indianapolis, IN, USA). The animals were housed in a protected unit for immunodeficient animals with 12-h light–dark cycles and provided with sterilized food and water ad libitum. When tumor volume reached an average size of 150 mm^3^, the animals were randomized into two groups: control (n = 5), PDMP (n = 7) treated mice. PDMP was dissolved in 5% of Tween 80%–0.9% NaCl and then 100 µL of 1:10 dilution in 0.9% NaCl (equivalent to 5 mg/kg dose) was intratumorally administered every day. Considering that all in vitro experiments with OR cells were performed in the presence of osimertinib, all mice were treated daily with osimertinib (3 mg/kg; oral gavage). Tumor xenografts were measured as previously described^49^. After 22 days of treatment, mice were euthanized by carbon dioxide inhalation.

All procedures adhered to the ARRIVE guidelines (https://arriveguidelines.org), which are grounded in the Ethical Principle in Animal Research endorsed by the Local Ethical Committee of University of Parma (Organismo per la Protezione e il Benessere degli Animali) and by the Italian Ministry of Health, in accordance with the institutional guidelines that are in compliance with national (D.Lgs. 26/2014) and international (Directive 2010/63/EU) laws and policies. The protocol was officially registered under the number 430-2022-PR for animals utilized in experimentation.

### Statistical analysis

GraphPad Prism version 8.0 software (GraphPad Software Inc., San Diego, CA, USA) was used for data analysis. Results are expressed as mean values ± standard deviations (SD). Differences between the mean values recorded for different experimental conditions were evaluated by Student’s *t*-test and *p* values are indicated where appropriate in the figures and their legends. For in vivo studies comparison among groups was made using two-way repeated measures ANOVA followed by Bonferroni’s post-test (to adjust for multiple comparisons). *p* values of less than 0.05 were considered significant.

### Supplementary Information

Supplementary Information.

## Data Availability

All data generated or analyzed during this study are included in this published article (and its supplementary information files).
