# Supplementary material for: Targeting glucosylceramide synthase induces antiproliferative and proapoptotic effects in osimertinib-resistant NSCLC cell models
Source: Sci Rep. 2024 Mar 18;14:6491. doi: 10.1038/s41598-024-57028-8 (PMC10948837; doi:10.1038/s41598-024-57028-8)
Supplement: Supplementary file 1 — Supplementary Information. [file 41598_2024_57028_MOESM1_ESM.pdf]

Supplementary Materials for

Targeting Glucosylceramide Synthase induces antiproliferative and proapoptotic effects in osimertinib-resistant NSCLC cell models.

Silvia La Monica[1], Federica Vacondio[2], Kamal Eltayeb [1], Alessio Lodola[2], Francesco Volta[1], Martina Viglioli[2], Francesca Ferlenghi[2], Francesca Galvani[2], Maricla Galetti[3], Mara Bonelli[1], Claudia Fumarola[1], Andrea Cavazzoni[1], Lisa Flammini[2], Michela Verzè[4], Roberta Minari[4], Pier Giorgio Petronini[1], Marcello Tiseo[1,4]*, Marco Mor[2] and Roberta Alfieri[1].

[1]Department of Medicine and Surgery, University of Parma, 43126 Parma, Italy;
[2]Department of Food and Drug, University of Parma, 43124 Parma, Italy.
[3]Department of Occupational and Environmental Medicine, Epidemiology and Hygiene, INAIL-Italian Workers' Compensation Authority, Monte Porzio Catone, 00078 Rome, Italy.
[4]Medical Oncology Unit, University Hospital of Parma, 43126 Parma, Italy;

*Correspondence: marcello.tiseo@unipr.it

The PDF File includes:

Table S1: Top-thirty identified lipids emerging from two-class PLS-DA on osimertinib-sensitive (OS) and osimertinib-resistant (OR) cell lines according to VIP values.

Table S2: Identified sphingolipids between PC9^{BRAFG469A} and PC9 cell lines.

Table S3: Identified sphingolipids between PC9T790M^{clA} and PC9T790M cell lines.

Table S4: Identified sphingolipids between PC9T790M^{clC} and PC9T790M cell lines.

Table S5: Identified sphingolipids between PC9T790M^{C797S} and PC9T790M cell lines.

Table S6: Identified sphingolipids PC9^{BRAFG469A} + PDMP and PC9^{BRAFG469A} cell lines.

Table S7: Identified sphingolipids PC9T790M^{clA} + PDMP and PC9T790M^{clA} cell lines.

Table S8: Identified sphingolipids between PC9T790M^{clC} + PDMP and PC9T790M^{clC} cell lines.

Table S9: Identified sphingolipids between PC9T790M^{C797S} + PDMP and PC9T790M^{C797S} cell lines.

Figure S1: Change of putatively identified sphingolipids between PC9T790M^{clC} and PC9T790M performed in two different days (inter-day repeatability).

Figure S2: Osimertinib resistant cell lines treated with increasing concentrations of eliglustat or PDMP.

Figure S3: Original western blot images included in Figure 3E. Cropped western blot bands are highlighted by red boxes.

Figure S4: Original western blot images included in Figure 6B. Cropped western blot bands are highlighted by red boxes.

Table S1. Top-thirty identified lipids emerging from two-class PLS-DA on osimertinib-sensitive (OS) and osimertinib-resistant (OR) cell lines according to VIP values. Lipid categories (fatty acyls, FA; glycerophospholipids, GP; glycerolipids, GL; sphingolipids, SP), classes and lipid identities are annotated according to their abbreviation in LIPID MAPS Structure Database (LMSD) Classification System.

**Lipid Category**	**Lipid Class**	**Lipid ID**	**Formula**	**Observed m/z**	**Adducts**	**Mass Error (ppm)**	**RT (min)**	**CCS (Å^2)**	**VIP**
**SP**	*Acidic glycosphingolipids*	NeuAcHex2Cer 34:1;O2	C57H104N2O21	1153.7216	M+H	1.01	6.09	362.3	2.9
GP	*Glycerophosphocholines*	PC 34:2	C42H80NO7P	764.5555	M+Na	-1.32	8.33	297.2	2.9
GL	*Triradylglycerols*	TG 60:9	C63H104O6	974.8160	M+NH4	-1.13	13.45	355.8	2.6
**SP**	*Neutral glycosphingolipids*	Hex2HexNAc-Cer 34:1;O2	C54H100N2O18	1087.6870	M+Na	0.63	7.11	345.1	2.3
**SP**	*Phosphosphingolipids*	SM 34:0;O2	C39H81N2O6P	727.5726	M+Na	0.13	7.70	303.7	2.3
**SP**	*Neutral glycosphingolipids*	Hex2Cer 38:1;O2	C50H95NO13	940.6704	M+H, M+Na, M+H-H2O	0.84	9.00	332.3	2.2
**SP**	*Neutral glycosphingolipids*	Hex2Cer 44:1;O2	C56H107NO13	1002.7810	M+H	-0.48	10.89	346.3	2.2
**SP**	*Neutral glycosphingolipids*	Hex2Cer 43:1;O2	C55H105NO13	988.7642	M+H	-1.69	10.60	346.5	2.2
GL	*Triradylglycerols*	TG 62:12	C65H102O6	996.8007	M+NH4	-0.75	13.04	355.4	2.1
GL	*Triradylglycerols*	TG 54:5	C57H102O5	867.7804	M+H	0.46	14.16	336.5	2.1
FA	*	*	C35H66O4	551.5032	M+H	-0.23	7.00	270.3	2.1
FA	*Fatty esters*	WE 34:3	C34H62O2	520.5077	M+NH4	-2.12	7.81	262.7	2.0
FA	*Fatty amides*	**	C37H70N2O7	637.5152	M+H-H2O	0.33	7.22	276.8	1.9
GP	*Glycerophosphoglycerols*	PG O-39:1	C45H89O9P	805.6330	M+H	1.71	9.42	308.1	1.9
GL	*Triradylglycerols*	TG 52:6	C55H94O6	868.7387	M+NH4, M+Na	-0.16	12.82	327.6	1.9
GL	*Triradylglycerols*	TG 52:1	C55H106O5	869.7914	M+Na	-2.20	14.40	339.4	1.9
**SP**	*Neutral glycosphingolipids*	HexCer 42:2;O2	C48H91NO8	792.6706	M+H-H2O	-0.73	10.26	317.1	1.9
GL	*Triradylglycerol*	TG 58:2	C61H114O6	960.8961	M+NH4	0.80	14.52	359.0	1.8
**SP**	*Neutral glycosphingolipids*	Hex2Cer 36:1;O2	C48H91NO13	912.6390	M+H-H2O, M+Na, M+K, M+H	0.86	8.23	326.9	1.8
GL	*Triradylglycerols*	TG 56:9	C59H96O6	918.7529	M+NH4	-1.84	12.72	338.6	1.8
GL	*Triradylglycerols*	TG 56:4	C59H106O6	928.8315	M+NH4, M+Na	-1.38	13.98	353.5	1.7
**SP**	*Neutral glycosphingolipids*	HexCer 44:1;O2	C50H97NO8	862.7101	M+H, M+Na, M+H-H2O	-0.66	11.18	327.7	1.7
FA	*fatty esters*	FAHFA 35:3;O	C35H62O4	547.4716	M+H	-0.82	9.29	253.6	1.7
**SP**	*Ceramides*	Cer 42:1;O2	C42H83NO3	650.6442	M+H, M+Na, M+K	-0.47	11.03	293.7	1.7
**SP**	*Neutral glycosphingolipids*	HexCer 41:2;O2	C47H89NO8	818.6486	M+H-H2O, M+Na	0.76	9.73	313.7	1.6
**SP**	*Ceramides*	Cer 42:2;O2	C42H81NO3	630.6178	M+H-H2O	-0.81	9.69	288.4	1.6
GL	*Triradylglycerols*	TG 54:7	C57H96O6	894.7540	M+NH4, M+Na	-0.60	12.96	336.0	1.6
**SP**	*Phosphosphingolipids*	SM 40:0;O2	C45H93N2O6P	789.6838	M+H	-0.82	10.00	323.0	1.6
GP	*Glycerophosphoinositols*	PI 32:1	C41H77O13P	809.5178	M+H, M+Na, M+K	0.45	6.39	302.2	1.6
GL	*Diradylglycerols*	DG O-34:1	C37H72O4	603.5328	M+Na	0.78	13.44	271.9	1.6

*Annotated as *Artemoin A* by Human Metabolome Database (HMDB); **Annotated as *Flavolipin* by LMSD.

Table S2. Identified sphingolipids (lipid ID, formula, observed m/z, adducts, mass error (ppm), retention time (RT, min), collisional cross section (CCS, $Å^2$), average log_2 intensity ± standard deviation, p-value, and fold change (log_2) between $PC9^{BRAFG469A}$ and PC9 cell lines.

Lipid ID	Formula	Observed m/z[a]	Adducts[b]	Mass Error (ppm)[a]	RT (min)[a]	CCS ($Å^2$)[a]	$PC9^{BRAFG469A}$ (ave. ± SD)	PC9 (ave. ± SD)	p-value[c]	Fold change (log_2) $PC9^{BRAFG469A}$ *vs* PC9
SM 42:2;O2	C47H93N2O6P	835.6663	M+Na	-0.02	10.00	322.5	6.85 ± 0.43	8.55 ± 0.15	3.06E-04	-1.70
SM 42:1;O2	C47H95N2O6P	837.6807	M+Na	-1.62	10.06	325.8	7.11 ± 0.25	5.97 ± 0.14	1.98E-04	1.15
SM 42:1;O2	C47H95N2O6P	853.6578	M+K	2.36	10.45	328.8	6.51 ± 0.37	7.17 ± 0.18	1.75E-02	-0.66
SM 42:0;O2	C47H97N2O6P	817.7155	M+H, M+Na	-0.23	10.66	329.5	11.72 ± 0.4	9.64 ± 0.11	5.98E-05	2.08
SM 30:1;O2	C35H71N2O6P	647.5129	M+H, M+Na	1.04	5.64	287.2	11.6 ± 0.09	10.37 ± 0.01	1.89E-07	1.23
SM 34:2;O3	C39H77N2O7P	717.5545	M+H	0.51	6.46	301.8	6.75 ± 0.1	6.38 ± 0.04	4.42E-04	0.37
SM 34:2;O2	C39H77N2O6P	701.5600	M+H, M+Na	1.16	6.80	295.6	11.08 ± 0.08	10.96 ± 0.05	5.00E-02	0.12
SM 34:1;O2	C39H79N2O6P	725.5577	M+Na, M+K, M+H-H2O	1.25	7.48	301.6	12.61 ± 0.07	11.08 ± 0.1	2.94E-07	1.53
SM 34:0;O2	C39H81N2O6P	705.5914	M+H, M+Na	1.25	7.80	305.3	13.35 ± 0.03	11.67 ± 0.03	2.41E-10	1.69
SM 35:1;O2	C40H81N2O6P	717.5905	M+H, M+Na	0.03	7.93	305.1	9.85 ± 0.15	9.74 ± 0.08	2.39E-01	0.11
SM 36:1;O2	C41H83N2O6P	753.5887	M+Na	0.88	8.32	307.6	10.04 ± 0.1	8.78 ± 0.15	8.63E-06	1.26
SM 38:1;O2	C43H87N2O6P	759.6379	M+H, M+Na	0.64	9.10	314.0	11.19 ± 0.12	12.48 ± 0.06	1.30E-06	-1.29
SM 40:2;O2	C45H89N2O6P	807.6352	M+Na	0.15	9.32	316.4	6.77 ± 0.16	6.17 ± 0.35	2.15E-02	0.59
SM 42:3;O2	C47H91N2O6P	811.6696	M+Na, M+K, M+H	0.96	9.32	319.6	11.89 ± 0.2	12.25 ± 0.04	1.30E-02	-0.36
SM 40:1;O2	C45H91N2O6P	825.6252	M+K	0.73	9.80	319.3	4.19 ± 0.36	5.63 ± 0.12	2.73E-04	-1.44
SM	C45H91N2O6P	809.6514	M+Na	0.89	9.81	319.6	10.05 ± 0.13	10.42 ± 0.07	2.54E-03	-0.37

40:1;O2										
SM 42:2;O2	C47H93N2O6P	835.6671	M+Na, M+K	0.93	9.82	322.5	10.85 ± 0.11	11.21 ± 0.12	5.09E-03	-0.36
Cer 42:2;O2	C42H81NO3	630.6176	M+H-H2O, M+H	-1.22	10.16	290.8	10.23 ± 0.1	8.92 ± 0.08	7.02E-07	1.31
Cer 42:0;O3	C42H85NO4	650.6429	M+H-H2O	-2.54	10.38	296.8	8.74 ± 0.04	5.29 ± 0.18	2.33E-08	3.44
Cer 42:0;O3	C42H85NO4	650.6432	M+H-H2O	-2.16	10.42	346.7	5.51 ± 0.52	2.75 ± 0.21	6.15E-05	2.76
Cer 40:0;O2	C40H81NO3	646.6112	M+Na	0.61	10.49	290.4	2.19 ± 0.42	5.61 ± 0.18	5.59E-06	-3.42
Cer 42:1;O3	C42H83NO4	648.6277	M+H-H2O	-1.79	10.57	296.8	6.46 ± 0.57	6.46 ± 0.27	9.89E-01	0.00
Cer 42:1;O3	C42H83NO4	666.6395	M+H	0.10	10.57	322.7	3.05 ± 0.87	3.24 ± 0.42	7.06E-01	-0.19
Cer 42:2;O	C42H81NO2	614.6230	M+H-H2O	-0.70	10.76	288.0	7.91 ± 0.28	6.02 ± 0.13	1.85E-05	1.89
Cer 42:1;O2	C42H83NO3	632.6339	M+H-H2O, M+H	-0.07	10.76	294.0	11.94 ± 0.25	10.06 ± 0.07	7.29E-06	1.88
Cer 40:1;O2	C40H79NO3	604.6029	M+H-H2O, M+Na, M+H	0.23	10.92	288.2	10.74 ± 0.34	11.05 ± 0.17	1.57E-01	-0.31
Cer 42:0;O2	C42H85NO3	652.6607	M+H-H2O, M+H	0.70	10.94	300.0	10.75 ± 0.47	6.18 ± 0.09	1.40E-06	4.57
Cer 42:2;O2	C42H81NO3	630.6185	M+H-H2O, M+Na, M+H	0.26	11.07	290.8	9.79 ± 0.08	10.91 ± 0.1	2.23E-06	-1.13
Cer 40:0;O2	C40H81NO3	606.6179	M+H-H2O	-0.67	11.10	284.9	7.36 ± 0.55	6.49 ± 0.16	2.34E-02	0.87
Cer 40:0;O2	C40H81NO3	624.6289	M+H, M+Na	-0.01	11.11	290.9	10.14 ± 0.58	8.38 ± 0.25	1.33E-03	1.77
Cer 42:1;O2	C42H83NO3	650.6445	M+H, M+Na	-0.10	11.13	296.8	10.01 ± 0.04	9.06 ± 0.15	1.96E-05	0.95
Cer 42:1;O2	C42H83NO3	632.6344	M+H-H2O, M+K, M+H, M+Na	0.64	11.47	294.0	12.42 ± 0.79	11.85 ± 0.14	2.08E-01	0.57
Cer 42:2;O	C42H81NO2	614.6230	M+H-H2O	-0.63	11.48	291.2	6.28 ± 1.04	5.98 ± 0.11	5.81E-01	0.30
Cer 42:1;O	C42H83NO2	634.6492	M+H	-0.71	11.64	293.9	7.89 ± 0.85	7.07 ± 0.05	1.02E-01	0.82

Cer 42:0;O2	C42H85NO3	652.6605	M+H, M+Na	0.49	11.65	296.7	10.47 ± 1.13	8.16 ± 0.26	7.21E-03	2.31
Cer 42:0;O	C42H85NO2	636.6646	M+H	-1.05	12.02	297.1	4.2 ± 0.92	3.55 ± 0.4	2.42E-01	0.65
Cer 30:1;O2	C30H59NO3	464.4454	M+H-H2O	-1.58	5.02	257.3	6.23 ± 0.32	4.88 ± 0.24	4.87E-04	1.35
Cer 30:1;O2	C30H59NO3	464.4461	M+H-H2O, M+H	-0.26	5.75	251.2	7.27 ± 0.07	6.09 ± 0.07	3.96E-07	1.18
Cer 30:1;O2	C30H59NO3	464.4465	M+H-H2O, M+H	0.52	6.17	251.2	8.22 ± 0.06	6.97 ± 0.06	1.30E-07	1.25
Cer 30:1;O2	C30H59NO3	464.4466	M+H-H2O, M+K, M+Na	0.80	7.15	251.2	12.03 ± 0.04	11 ± 0.05	4.23E-08	1.03
Cer 32:1;O2	C32H63NO3	492.4768	M+H-H2O, M+Na	-1.42	8.03	256.5	7.95 ± 0.14	6.5 ± 0.14	6.15E-06	1.45
Cer 34:2;O2	C34H65NO3	518.4918	M+H-H2O	-2.58	8.22	258.9	5.35 ± 0.15	5.97 ± 0.13	7.19E-04	-0.63
Cer 34:1;O2	C34H67NO3	520.5090	M+H-H2O, M+K, M+Na	0.42	8.85	265.0	12.27 ± 0.11	11.53 ± 0.14	1.60E-04	0.74
Cer 34:0;O2	C34H69NO3	540.5349	M+H-H2O, M+H	-0.36	9.13	267.6	11.14 ± 0.13	9.16 ± 0.18	2.07E-06	1.99
Cer 35:1;O2	C35H69NO3	534.5235	M+H-H2O	-1.82	9.23	267.8	4.89 ± 0.09	4.34 ± 0.26	7.39E-03	0.55
Cer 40:1;O2	C40H79NO3	604.6024	M+H-H2O	-0.58	9.55	288.2	3.26 ± 0.49	3.17 ± 0.06	7.30E-01	0.09
Cer 36:1;O2	C36H71NO3	548.5400	M+H-H2O, M+K, M+Na	-0.21	9.61	273.7	10.3 ± 0.1	10.96 ± 0.18	6.53E-04	-0.65
Cer 40:0;O3	C40H81NO4	622.6116	M+H-H2O	-2.71	9.78	340.6	5.29 ± 0.31	3.86 ± 0.23	3.27E-04	1.43
HexCer 41:2;O2	C47H89NO8	796.6663	M+H	0.27	10.26	319.9	5 ± 0.69	6.41 ± 0.08	6.53E-03	-1.41
HexCer 44:2;O2	C50H95NO8	860.6940	M+Na	-1.22	10.73	328.7	7.38 ± 0.11	6.4 ± 0.16	5.55E-05	0.98
HexCer 42:2;O2	C48H91NO8	832.6644	M+H-H2O, M+Na, M+H	0.78	10.16	319.2	14.47 ± 0.06	13.29 ± 0.06	1.80E-07	1.18
HexCer 42:2;O2	C48H91NO8	810.6809	M+H	-1.10	10.31	319.6	10.13 ± 0.12	9.19 ± 0.06	7.13E-06	0.94
HexCer 40:0;O2	C46H91NO8	786.6821	M+H, M+Na	0.37	10.35	320.1	12.26 ± 0.3	8.79 ± 0.07	5.24E-07	3.47

HexCer 42:1;O3	C48H93NO9	810.6824	M+H-H2O, M+Na, M+H	0.80	10.55	322.9	11.24 ± 0.52	11.06 ± 0.21	5.35E-01	0.18
HexCer 42:1;O2	C48H93NO8	850.6511	M+K	-2.65	10.76	322.2	10.06 ± 0.26	8.58 ± 0.05	2.98E-05	1.48
HexCer 43:2;O3	C49H93NO9	857.7168	M+NH4	-2.46	11.14	328.7	2.94 ± 0.53	4.53 ± 0.24	1.58E-03	-1.59
HexCer 34:1;O2	C40H77NO8	682.5616	M+H-H2O, M+H	-0.14	6.34	296.1	6.8 ± 0.27	5.12 ± 0.3	1.76E-04	1.68
HexCer 42:0;O2	C48H95NO8	836.6952	M+Na	0.28	10.94	322.4	11.38 ± 0.47	7.04 ± 0.1	1.84E-06	4.34
Hex2Cer 36:1;O2	C48H91NO13	912.6387	M+Na	0.44	8.40	327.7	10.72 ± 0.24	6.61 ± 0.32	8.45E-07	4.11
Hex2Cer 40:0;O2	C52H101NO13	948.7350	M+H, M+Na	0.41	10.00	347.3	11.59 ± 0.33	7.75 ± 0.05	4.68E-07	3.83
Hex2Cer 44:2;O2	C56H105NO13	1000.7634	M+H	-2.52	10.40	349.9	7.91 ± 0.22	6.5 ± 0.14	3.58E-05	1.41
Hex2Cer 42:1;O2	C54H103NO13	974.7516	M+H, M+Na, M+H-H2O	1.44	10.41	343.5	14.68 ± 0.29	12.85 ± 0.07	1.72E-05	1.83
Hex2Cer 42:0;O2	C54H105NO13	976.7664	M+H, M+Na	0.54	10.60	350.3	11.69 ± 0.47	6.94 ± 0.24	1.78E-06	4.75
Hex2Cer 44:1;O2	C56H107NO13	1002.7824	M+H	0.91	10.97	353.2	8.3 ± 0.5	6.06 ± 0.24	1.98E-04	2.25
Hex2Cer 30:1;O2	C42H79NO13	806.5635	M+H, M+Na, M+H-H2O	1.37	5.76	303.3	11.45 ± 0.09	10.24 ± 0.01	1.61E-07	1.20
Hex2Cer 32:1;O2	C44H83NO13	834.5951	M+H, M+Na, M+H-H2O	1.63	6.67	312.6	7.88 ± 0.12	5.84 ± 0.06	1.04E-07	2.04
Hex2Cer 34:1;O2	C46H87NO13	862.6257	M+H, M+Na, M+K, M+H-H2O	0.83	7.55	322.0	13.45 ± 0.08	10.81 ± 0.07	4.65E-09	2.64
Hex2Cer 36:1;O2	C48H91NO13	890.6566	M+H-H2O, M+H	0.33	8.37	328.1	10.95 ± 0.08	9.32 ± 0.07	6.03E-08	1.63
Hex2Cer 38:1;O2	C50H95NO13	940.6705	M+H, M+Na, M+H-H2O	0.97	9.11	333.9	10.25 ± 0.15	10.15 ± 0.09	2.85E-01	0.10
Hex2Cer 40:1;O2	C52H99NO13	946.7200	M+Na, M+K, M+H, M+H-H2O	1.14	9.78	340.6	13.68 ± 0.17	12.5 ± 0.1	2.17E-05	1.19
Hex2Cer 42:2;O2	C54H101NO13	972.7355	M+H, M+Na, M+H-H2O	0.98	9.80	343.5	13.86 ± 0.09	12.19 ± 0.14	9.75E-07	1.67
Hex2Cer 42:2;O2	C54H101NO13	972.7337	M+H, M+Na	-0.94	9.98	343.5	9.61 ± 0.33	8.8 ± 0.18	5.27E-03	0.81

Hex2Cer 43:1;O2	C55H105NO13	988.7666	M+H	0.75	10.70	350.1	6.99 ± 0.47	4.63 ± 0.18	8.65E-05	2.37
Hex3Cer 42:1;O2	C60H113NO18	1136.8042	M+H, M+Na, M+H-H2O	0.97	10.19	368.3	9.25 ± 0.48	8.86 ± 0.38	2.41E-01	0.40
Hex3Cer 34:1;O2	C52H97NO18	1024.6796	M+H, M+Na, M+H-H2O	1.65	7.28	342.7	8.77 ± 0.23	8.92 ± 0.14	3.16E-01	-0.15
Hex3Cer 40:1;O2	C58H109NO18	1108.7728	M+H-H2O, M+Na, M+H	0.90	9.55	361.8	8.2 ± 0.35	9.43 ± 0.09	5.12E-04	-1.22
Hex3Cer 42:2;O2	C60H111NO18	1134.7881	M+H, M+Na, M+H-H2O	0.58	9.57	364.9	9.25 ± 0.35	9.74 ± 0.18	4.82E-02	-0.49

[a]*Property reported for the most abundant ion;* [b]*If more ions are found, the most abundant is the first one reported.* [c]*Statistical significance was set at p<0.05 by unpaired two-tailed t-test (n=4 per cell line). Expressed in scientific notation.*

Table S3. Identified sphingolipids (Lipid ID, formula, observed m/z, adducts, mass error (ppm), retention time (RT, min), collisional cross section (CCS, Å^2), average log_2 intensity ± standard deviation, p-value, and fold change (log_2) between PC9T790M[clA] and PC9T790M cell lines.

Lipid ID	Formula	Observed m/z[a]	Adducts[b]	Mass Error (ppm)[a]	RT (min)[a]	CCS (Å^2)[a]	PC9T790M[clA] (ave. ± SD)	PC9T790M (ave. ± SD)	p-value[c]	Fold change (log_2) PC9T790M[clA] *vs* PC9T790M
SM 32:2;O2	C37H73N2O6P	673.5271	M+H	-1.25	5.78	287.9	5.14 ± 0.71	5.27 ± 0.65	8.18E-01	-0.13
SM 32:1;O2	C37H75N2O6P	675.5441	M+H, M+Na	0.90	6.54	291.0	12.06 ± 0.3	12.22 ± 0.23	4.68E-01	-0.17
SM 34:1;O2	C39H79N2O6P	725.5576	M+Na, M+K	1.14	7.46	299.3	13.77 ± 0.18	13.92 ± 0.24	3.92E-01	-0.15
SM 34:0;O2	C39H81N2O6P	705.5911	M+H	0.74	7.79	302.9	13.6 ± 0.31	13.04 ± 0.35	7.33E-02	0.56
SM 40:2;O2	C45H89N2O6P	807.6339	M+Na	-1.46	9.29	313.5	6.61 ± 0.45	7.51 ± 0.8	1.13E-01	-0.90
SM 42:3;O2	C47H91N2O6P	811.6690	M+H, M+Na	0.28	9.30	316.6	12.15 ± 0.56	12.85 ± 0.28	1.08E-01	-0.70
SM 40:1;O2	C45H91N2O6P	787.6691	M+H	0.43	9.82	317.1	13.52 ± 0.43	13.97 ± 0.25	1.66E-01	-0.45
SM 42:2;O2	C47H93N2O6P	813.6851	M+H	0.91	9.82	323.0	14.83 ± 0.3	15 ± 0.18	4.29E-01	-0.17
SM 40:1;O2	C45H91N2O6P	809.6506	M+Na	-0.19	9.83	319.9	8.46 ± 1.1	8.5 ± 1.04	9.63E-01	-0.04
SM 42:2;O2	C47H93N2O6P	813.6850	M+H, M+Na	0.75	10.00	319.8	12.8 ± 0.31	12.95 ± 0.18	4.82E-01	-0.15
SM 42:1;O2	C47H95N2O6P	837.6822	M+Na, M+K	0.30	10.45	322.5	11.12 ± 0.57	11 ± 0.36	7.48E-01	0.13
SM 42:0;O2	C47H97N2O6P	817.7157	M+H, M+Na	-0.03	10.66	326.1	12.11 ± 0.41	11.24 ± 0.33	3.13E-02	0.87
SM 44:1;O2	C49H99N2O6P	843.7304	M+H	-1.09	11.04	328.8	6.35 ± 1.18	7.68 ± 0.49	1.31E-01	-1.33
Cer 34:2;O2	C34H65NO3	518.4918	M+H-H2O, M+Na	-2.64	8.15	260.7	5.85 ± 0.12	5.17 ± 0.29	7.52E-03	0.68
Cer 34:0;O2	C34H69NO3	562.5170	M+Na	-0.07	9.06	265.7	9.21 ± 0.39	9.19 ± 0.38	9.43E-01	0.02

Cer 36:1;O2	C36H71NO3	588.5330	M+H-H2O, M+Na	0.69	9.55	271.2	10.62 ± 0.45	11.01 ± 0.37	2.76E-01	-0.39
Cer 42:1;O2	C42H83NO3	632.6327	M+H-H2O, M+H	-2.00	10.73	295.0	10.1 ± 0.5	10.45 ± 0.33	3.48E-01	-0.35
Cer 40:1;O2	C40H79NO3	604.6024	M+H-H2O	-0.53	10.86	289.4	9.86 ± 0.45	10.32 ± 0.27	1.80E-01	-0.46
Cer 42:2;O2	C42H81NO3	630.6185	M+H-H2O, M+H, M+Na	0.16	10.88	292.0	12.11 ± 0.28	12.33 ± 0.17	2.86E-01	-0.22
Cer 48:2;O2	C48H93NO3	749.7489	M+NH4	-0.67	11.01	321.0	4.27 ± 1.05	3.36 ± 1.38	3.63E-01	0.91
Cer 42:0;O3	C42H85NO4	650.6436	M+H-H2O	-1.42	11.08	294.6	8.67 ± 0.57	8.14 ± 0.37	2.22E-01	0.53
Cer 41:1;O2	C41H81NO3	618.6174	M+H-H2O, M+Na	-1.55	11.16	292.2	7.15 ± 0.53	7.55 ± 0.34	3.06E-01	-0.40
Cer 42:1;O2	C42H83NO3	632.6341	M+H-H2O, M+H, M+K, M+Na	0.21	11.43	295.0	12.21 ± 0.42	12.64 ± 0.2	1.62E-01	-0.43
HexCer 40:1;O2	C46H89NO8	806.6474	M+H-H2O, M+Na, M+H	-0.83	10.12	313.5	13.21 ± 0.47	13.74 ± 0.33	1.55E-01	-0.53
HexCer 42:2;O2	C48H91NO8	832.6638	M+NH4, M+Na, M+K, M+H, M+H-H2O	0.07	10.13	316.2	13.76 ± 0.37	14.14 ± 0.27	1.96E-01	-0.38
HexCer 42:2;O2	C48H91NO8	832.6635	M+H-H2O, M+Na, M+H	-0.25	10.29	316.2	10.96 ± 0.52	10.97 ± 0.4	9.80E-01	-0.01
HexCer 41:1;O2	C47H91NO8	820.6633	M+H, M+Na, M+H-H2O	-0.51	10.43	316.5	10.37 ± 0.67	10.67 ± 0.48	5.40E-01	-0.30
HexCer 42:1;O2	C48H93NO8	812.6978	M+H, M+K, M+Na, M+H-H2O	0.47	10.74	319.8	14.53 ± 0.4	14.8 ± 0.24	3.50E-01	-0.27
HexCer 42:0;O2	C48H95NO8	814.7120	M+H, M+Na	-1.25	10.92	323.0	10.55 ± 0.6	9.21 ± 0.39	2.13E-02	1.33
Hex2Cer 34:1;O2	C46H87NO13	862.6254	M+H-H2O, M+H	0.44	7.52	318.9	13.55 ± 0.21	12.85 ± 0.28	1.21E-02	0.70
Hex2Cer 36:1;O2	C48H91NO13	890.6561	M+H-H2O, M+H	-0.29	8.35	324.8	11.37 ± 0.26	10.82 ± 0.26	3.98E-02	0.55
Hex2Cer 32:0;O2	C44H85NO13	818.5984	M+H-H2O	-0.48	8.93	310.2	12.52 ± 0.29	11.72 ± 0.24	1.23E-02	0.80

Hex2Cer 40:1;O2	C52H99NO13	946.7191	M+H	0.13	9.80	336.7	13.06 ± 0.38	11.91 ± 0.47	1.52E-02	1.15
Hex2Cer 42:2;O2	C54H101NO13	972.7346	M+H	0.04	9.82	339.5	13.35 ± 0.28	12.23 ± 0.38	6.42E-03	1.12
Hex2Cer 42:2;O2	C54H101NO13	972.7338	M+H	-0.84	9.96	339.5	11.09 ± 0.41	9.6 ± 0.5	7.35E-03	1.48
Hex2Cer 40:0;O2	C52H101NO13	948.7351	M+H, M+Na	0.53	10.00	343.1	11.53 ± 0.54	8.93 ± 0.62	1.92E-03	2.60
Hex2Cer 42:0;O2	C54H105NO13	976.7663	M+H, M+Na	0.45	10.61	345.9	11.54 ± 0.53	8.79 ± 0.67	1.70E-03	2.75
Hex2Cer 36:1;O2	C48H91NO13	912.6380	M+Na	-0.30	8.37	327.6	11.78 ± 0.09	11.04 ± 0.06	6.57E-05	0.74
Hex3Cer 34:1;O2	C52H97NO18	1024.6784	M+H, M+Na	0.51	7.27	338.7	6.83 ± 0.77	8.86 ± 0.43	1.00E-02	-2.03
Hex3Cer 34:1;O2	C52H97NO18	1006.6674	M+H-H2O	0.07	7.29	335.7	-1.35 ± 2.95	2.89 ± 0.22	6.02E-02	-4.23
Hex3Cer 40:1;O2	C58H109NO18	1108.7719	M+H, M+Na	0.06	9.56	356.9	4.35 ± 1.04	7.92 ± 0.26	2.37E-03	-3.57
Hex3Cer 42:2;O2	C60H111NO18	1134.7876	M+H-H2O, M+H	0.13	9.57	359.8	6.09 ± 1.04	8.5 ± 0.19	1.15E-02	-2.42
Hex3Cer 42:2;O2	C60H111NO18	1156.7691	M+Na	-0.21	9.57	359.5	2.41 ± 0.82	2.71 ± 1.56	7.52E-01	-0.30
Hex3Cer 42:1;O2	C60H113NO18	1136.8034	M+H-H2O, M+H, M+Na	0.33	10.19	363.1	7.6 ± 0.81	9.65 ± 0.26	9.17E-03	-2.05

[a]Property reported for the most abundant ion; [b]If more ions are found, the most abundant is the first one reported. [c]Statistical significance was set at $p<0.05$ by unpaired two-tailed t-test (n=4 and n=3 for PC9T790M^{clA} and PC9T790M cell lines, respectively). Expressed in scientific notation.

Table S4. Identified sphingolipids (lipid ID, formula, observed m/z, adducts, mass error (ppm), retention time (RT, min), collisional cross section (CCS, Å^2), average $\log_2$ intensity ± standard deviation, p-value, and fold change ($\log_2$) between PC9T790M^{clC} and PC9T790M cell lines.

Lipid ID	Formula	Observed m/z[a]	Adducts[b]	Mass Error (ppm)[a]	RT (min)[a]	CCS (Å^2)[a]	PC9T790M^{clC} (ave. ± SD)	PC9T790M (ave. ± SD)	p-value[c]	Fold change ($\log_2$) PC9T790M^{clC} *vs* PC9T790M
SM 32:2;O2	C37H73N2O6P	673.5271	M+H	-1.25	5.78	287.9	6.54 ± 0.97	5.27 ± 0.65	1.09E-01	1.27
SM 32:1;O2	C37H75N2O6P	675.5441	M+H, M+Na	0.90	6.54	291.0	12.01 ± 0.42	12.22 ± 0.23	4.70E-01	-0.21
SM 34:1;O2	C39H79N2O6P	725.5576	M+Na, M+K	1.14	7.46	299.3	13.72 ± 0.29	13.92 ± 0.24	3.60E-01	-0.21
SM 34:0;O2	C39H81N2O6P	705.5911	M+H	0.74	7.79	302.9	14.63 ± 0.56	13.04 ± 0.35	8.15E-03	1.58
SM 40:2;O2	C45H89N2O6P	807.6339	M+Na	-1.46	9.29	313.5	8.46 ± 0.96	7.51 ± 0.8	2.26E-01	0.95
SM 42:3;O2	C47H91N2O6P	811.6690	M+H, M+Na	0.28	9.30	316.6	13.68 ± 0.6	12.85 ± 0.28	8.11E-02	0.83
SM 40:1;O2	C45H91N2O6P	787.6691	M+H	0.43	9.82	317.1	14.01 ± 0.41	13.97 ± 0.25	8.98E-01	0.04
SM 42:2;O2	C47H93N2O6P	813.6851	M+H	0.91	9.82	323.0	14.86 ± 0.4	15 ± 0.18	6.06E-01	-0.14
SM 40:1;O2	C45H91N2O6P	809.6506	M+Na	-0.19	9.83	319.9	8.96 ± 1.63	8.5 ± 1.04	6.90E-01	0.46
SM 42:2;O2	C47H93N2O6P	813.6850	M+H, M+Na	0.75	10.00	319.8	14.58 ± 0.37	12.95 ± 0.18	9.39E-04	1.63
SM 42:1;O2	C47H95N2O6P	837.6822	M+Na, M+K	0.30	10.45	322.5	11.69 ± 0.66	11 ± 0.36	1.66E-01	0.69
SM 42:0;O2	C47H97N2O6P	817.7157	M+H, M+Na	-0.03	10.66	326.1	12.83 ± 0.33	11.24 ± 0.33	1.47E-03	1.59
SM 44:1;O2	C49H99N2O6P	843.7304	M+H	-1.09	11.04	328.8	7.94 ± 1.1	7.68 ± 0.49	7.21E-01	0.26
Cer 34:2;O2	C34H65NO3	518.4918	M+H-H2O, M+Na	-2.64	8.15	260.7	9.54 ± 0.33	5.17 ± 0.29	9.50E-06	4.37
Cer 34:0;O2	C34H69NO3	562.5170	M+Na	-0.07	9.06	265.7	8.84 ± 0.23	9.19 ± 0.38	1.91E-01	-0.35

Cer 36:1;O2	C36H71NO3	588.5330	M+H-H2O, M+Na	0.69	9.55	271.2	9.38 ± 0.58	11.01 ± 0.37	8.36E-03	-1.62
Cer 42:1;O2	C42H83NO3	632.6327	M+H-H2O, M+H	-2.00	10.73	295.0	9.35 ± 0.67	10.45 ± 0.33	4.92E-02	-1.10
Cer 40:1;O2	C40H79NO3	604.6024	M+H-H2O	-0.53	10.86	289.4	9.39 ± 0.46	10.32 ± 0.27	2.85E-02	-0.93
Cer 42:2;O2	C42H81NO3	630.6185	M+H-H2O, M+H, M+Na	0.16	10.88	292.0	11.39 ± 0.49	12.33 ± 0.17	2.68E-02	-0.94
Cer 48:2;O2	C48H93NO3	749.7489	M+NH4	-0.67	11.01	321.0	7.71 ± 0.73	3.36 ± 1.38	2.75E-03	4.35
Cer 42:0;O3	C42H85NO4	650.6436	M+H-H2O	-1.42	11.08	294.6	8.57 ± 0.79	8.14 ± 0.37	4.38E-01	0.42
Cer 41:1;O2	C41H81NO3	618.6174	M+H-H2O, M+Na	-1.55	11.16	292.2	7.04 ± 1.04	7.55 ± 0.34	4.60E-01	-0.51
Cer 42:1;O2	C42H83NO3	632.6341	M+H-H2O, M+H, M+K, M+Na	0.21	11.43	295.0	12.09 ± 0.28	12.64 ± 0.2	3.30E-02	-0.55
HexCer 40:1;O2	C46H89NO8	806.6474	M+H-H2O, M+Na, M+H	-0.83	10.12	313.5	12.23 ± 0.64	13.74 ± 0.33	1.40E-02	-1.52
HexCer 42:2;O2	C48H91NO8	832.6638	M+NH4, M+Na, M+K, M+H, M+H-H2O	0.07	10.13	316.2	12.7 ± 0.61	14.14 ± 0.27	1.32E-02	-1.43
HexCer 42:2;O2	C48H91NO8	832.6635	M+H-H2O, M+Na, M+H	-0.25	10.29	316.2	12.24 ± 0.57	10.97 ± 0.4	2.26E-02	1.26
HexCer 41:1;O2	C47H91NO8	820.6633	M+H, M+Na, M+H-H2O	-0.51	10.43	316.5	8.97 ± 1.44	10.67 ± 0.48	1.13E-01	-1.70
HexCer 42:1;O2	C48H93NO8	812.6978	M+H, M+K, M+Na, M+H-H2O	0.47	10.74	319.8	13.96 ± 0.31	14.8 ± 0.24	1.17E-02	-0.85
HexCer 42:0;O2	C48H95NO8	814.7120	M+H, M+Na	-1.25	10.92	323.0	9.7 ± 0.6	9.21 ± 0.39	2.85E-01	0.48
Hex2Cer 34:1;O2	C46H87NO13	862.6254	M+H-H2O, M+H	0.44	7.52	318.9	11.94 ± 0.23	12.85 ± 0.28	4.99E-03	-0.91
Hex2Cer 36:1;O2	C48H91NO13	890.6561	M+H-H2O, M+H	-0.29	8.35	324.8	7.46 ± 0.24	10.82 ± 0.26	9.99E-06	-3.37
Hex2Cer 32:0;O2	C44H85NO13	818.5984	M+H-H2O	-0.48	8.93	310.2	12.77 ± 0.4	11.72 ± 0.24	1.08E-02	1.05

Hex2Cer 40:1;O2	C52H99NO13	946.7191	M+H	0.13	9.80	336.7	11.07 ± 0.39	11.91 ± 0.47	4.65E-02	-0.84
Hex2Cer 42:2;O2	C54H101NO13	972.7346	M+H	0.04	9.82	339.5	11.13 ± 0.52	12.23 ± 0.38	2.82E-02	-1.10
Hex2Cer 42:2;O2	C54H101NO13	972.7338	M+H	-0.84	9.96	339.5	11.17 ± 0.49	9.6 ± 0.5	9.07E-03	1.57
Hex2Cer 40:0;O2	C52H101NO13	948.7351	M+H, M+Na	0.53	10.00	343.1	9.84 ± 0.7	8.93 ± 0.62	1.34E-01	0.91
Hex2Cer 42:0;O2	C54H105NO13	976.7663	M+H, M+Na	0.45	10.61	345.9	10.24 ± 0.34	8.79 ± 0.67	1.23E-02	1.45
Hex2Cer 36:1;O2	C48H91NO13	912.6380	M+Na	-0.30	8.37	327.6	7.32 ± 0.7	11.04 ± 0.06	2.88E-04	-3.72
Hex3Cer 34:1;O2	C52H97NO18	1024.6784	M+H, M+Na	0.51	7.27	338.7	11.86 ± 0.56	8.86 ± 0.43	6.05E-04	3.00
Hex3Cer 34:1;O2	C52H97NO18	1006.6674	M+H-H2O	0.07	7.29	335.7	8.32 ± 0.73	2.89 ± 0.22	6.56E-05	5.43
Hex3Cer 40:1;O2	C58H109NO18	1108.7719	M+H, M+Na	0.06	9.56	356.9	11.61 ± 0.49	7.92 ± 0.26	7.98E-05	3.69
Hex3Cer 42:2;O2	C60H111NO18	1134.7876	M+H-H2O, M+H	0.13	9.57	359.8	11.54 ± 0.4	8.5 ± 0.19	7.46E-05	3.04
Hex3Cer 42:2;O2	C60H111NO18	1156.7691	M+Na	-0.21	9.57	359.5	9.69 ± 1.7	2.71 ± 1.56	2.63E-03	6.98
Hex3Cer 42:1;O2	C60H113NO18	1136.8034	M+H-H2O, M+H, M+Na	0.33	10.19	363.1	13.4 ± 0.25	9.65 ± 0.26	6.80E-06	3.75

[a]*Property reported for the most abundant ion;* [b]*If more ions are found, the most abundant is the first one reported.* [c]*Statistical significance was set at p<0.05 by unpaired two-tailed t-test (n=4 and n=3 for PC9T790M*^{clC} *and PC9T790M cell lines, respectively). Expressed in scientific notation.*

Table S5. Identified sphingolipids (lipid ID, formula, observed m/z, adducts, mass error (ppm), retention time (RT, min), collisional cross section (CCS, Å^2), average $\log_2$ intensity ± standard deviation, p-value, and fold change ($\log_2$) between PC9T790M^{C797S} and PC9T790M cell lines.

Lipid ID	Formula	Observed m/z[a]	Adducts[b]	Mass Error (ppm)[a]	RT (min)[a]	CCS (Å^2)[a]	PC9T790M^{C797S} (ave. ± SD)	PC9T790M (ave. ± SD)	p-value[c]	Fold change ($\log_2$) PC9T790M^{C797S} *vs* PC9T790M
SM 30:1;O2	C35H71N2O6P	647.5124	M+H, M+K, M+Na	0.21	5.63	284.5	13.73 ± 0.19	14.93 ± 0.04	1.85E-05	-1.20
SM 32:0;O2	C37H77N2O6P	677.5592	M+H, M+Na	0.04	6.95	298.8	12.17 ± 0.16	11.44 ± 0.19	1.04E-03	0.73
SM 32:1;O2	C37H75N2O6P	675.5439	M+Na, M+K, M+H	0.57	6.59	292.8	14.57 ± 0.19	15.09 ± 0.2	9.00E-03	-0.52
SM 32:2;O2	C37H73N2O6P	673.5276	M+H, M+Na	-0.44	5.83	286.9	10.4 ± 0.19	10.49 ± 0.19	5.32E-01	-0.09
SM 34:1;O2	C39H79N2O6P	725.5569	M+Na	0.17	7.46	300.9	12.27 ± 0.52	13.38 ± 0.28	9.09E-03	-1.11
SM 34:2;O2	C39H77N2O6P	739.5146	M+K	-0.72	6.82	297.6	8.59 ± 0.12	7.96 ± 0.27	5.62E-03	0.62
SM 34:2;O2	C39H77N2O6P	723.5424	M+Na	1.75	7.03	297.9	4.13 ± 0.34	4.36 ± 0.11	2.52E-01	-0.23
SM 35:1;O2	C40H81N2O6P	717.5902	M+H, M+Na	-0.46	7.94	304.1	11.64 ± 0.08	11.56 ± 0.21	5.14E-01	0.08
SM 40:1;O2	C45H91N2O6P	809.6511	M+Na	0.51	9.86	320.6	11.93 ± 0.17	13.66 ± 0.04	9.37E-07	-1.74
SM 41:1;O2	C46H93N2O6P	801.6844	M+H, M+Na	0.03	10.22	323.9	13.72 ± 0.11	14 ± 0.18	3.98E-02	-0.28
SM 42:0;O2	C47H97N2O6P	817.7151	M+H, M+Na	-0.74	10.76	329.8	14.05 ± 0.09	13.13 ± 0.13	2.65E-05	0.92
SM 42:1;O2	C47H95N2O6P	853.6581	M+K	2.72	10.55	326.0	9.64 ± 0.03	10.54 ± 0.13	8.99E-06	-0.91
SM 42:2;O2	C47H93N2O6P	851.6398	M+K	-0.55	9.88	323.0	4.99 ± 0.3	8.8 ± 0.08	2.84E-07	-3.81
SM 42:2;O2	C47H93N2O6P	835.6664	M+Na	0.04	9.88	323.3	11.9 ± 0.11	14.32 ± 0.07	2.89E-08	-2.42
SM 42:2;O2	C47H93N2O6P	835.6667	M+Na	0.40	10.08	320.2	13.12 ± 0.05	13.77 ± 0.15	2.04E-04	-0.65

SM 44:1;O2	C49H99N2O6P	843.7309	M+H, M+Na	-0.54	11.15	332.4	9.62 ± 0.11	10.53 ± 0.16	9.38E-05	-0.91
Cer 30:1;O2	C30H59NO3	464.4452	M+H-H2O, M+H	-2.17	5.74	251.1	6.5 ± 0.5	8.17 ± 0.1	6.16E-04	-1.67
Cer 30:1;O2	C30H59NO3	464.4458	M+H-H2O, M+H	-0.88	6.15	251.1	8.63 ± 0.25	9.9 ± 0.02	4.79E-05	-1.27
Cer 30:1;O2	C30H59NO3	464.4460	M+Na, M+K, M+H, M+H-H2O	-0.38	7.13	251.1	13.39 ± 0.16	14.72 ± 0.1	7.17E-06	-1.33
Cer 36:1;O2	C36H71NO3	548.5396	M+H-H2O, M+Na, M+H, M+K	-0.91	9.63	272.0	11.69 ± 0.24	13.95 ± 0.02	1.43E-06	-2.26
Cer 40:0;O2	C40H81NO3	624.6282	M+H, M+Na	-1.21	11.15	291.0	10.96 ± 0.24	11.31 ± 0.2	6.85E-02	-0.35
Cer 40:1;O2	C40H79NO3	604.6011	M+H-H2O, M+H	-2.59	10.18	288.4	8.2 ± 0.06	11.11 ± 0.06	6.58E-10	-2.92
Cer 40:1;O2	C40H79NO3	604.6023	M+Na, M+K, M+H, M+H-H2O	-0.65	10.93	288.4	12.69 ± 0.08	14.51 ± 0.1	1.59E-07	-1.82
Cer 40:2;O2	C40H77NO3	602.5865	M+H-H2O, M+Na, M+H	-0.94	10.49	282.5	11.68 ± 0.12	13.34 ± 0.24	1.57E-05	-1.65
Cer 41:1;O2	C41H81NO3	658.6100	M+Na	-1.34	11.25	287.2	11.13 ± 0.12	11.7 ± 0.08	2.25E-04	-0.56
Cer 42:0;O	C42H85NO2	636.6645	M+H, M+Na	-1.26	12.05	296.7	10.09 ± 0.09	9.66 ± 0.16	3.25E-03	0.43
Cer 42:0;O2	C42H85NO3	652.6601	M+H, M+Na, M+K	-0.26	11.69	299.4	13.42 ± 0.25	13.4 ± 0.21	8.86E-01	0.02
Cer 42:0;O3	C42H85NO4	650.6431	M+H-H2O	-2.22	10.77	320.8	8.31 ± 0.05	10.2 ± 0.08	1.39E-08	-1.90
Cer 42:0;O3	C42H85NO4	650.6438	M+H-H2O	-1.26	11.16	293.4	10.9 ± 0.12	10.86 ± 0.17	7.13E-01	0.04
Cer 42:1;O	C42H83NO2	656.6309	M+Na	-1.07	11.66	284.3	9.31 ± 0.1	9.29 ± 0.09	8.31E-01	0.01
Cer 42:1;O	C42H83NO2	634.6491	M+H-H2O, M+H	-0.84	11.68	290.7	12.01 ± 0.06	11.89 ± 0.11	9.39E-02	0.12
Cer 42:1;O2	C42H83NO3	650.6440	M+H	-0.83	11.29	293.4	10.94 ± 0.11	8.35 ± 0.16	2.09E-07	2.59
Cer 42:1;O2	C42H83NO3	672.6263	M+Na, M+K, M+H	-0.23	11.51	289.9	14.19 ± 0.11	15.31 ± 0.08	3.85E-06	-1.12

Cer 42:2;O	C42H81NO2	614.6227	M+H-H2O	-1.20	11.51	288.2	8.97 ± 0.11	10.49 ± 0.15	2.81E-06	-1.52
Cer 42:2;O	C42H81NO2	632.6332	M+H-H2O, M+H	-1.28	10.77	293.8	10.11 ± 0.07	12.15 ± 0.1	5.99E-08	-2.05
Cer 42:2;O2	C42H81NO3	648.6276	M+H-H2O, M+H	-2.01	10.59	296.4	10.18 ± 0.1	8.47 ± 0.17	2.46E-06	1.71
Cer 42:2;O2	C42H81NO3	630.6182	M+Na, M+K, M+H, M+H-H2O	-0.30	10.96	293.8	13.03 ± 0.06	15.22 ± 0.2	8.69E-07	-2.19
Cer 42:2;O2	C42H81NO3	630.6182	M+H-H2O, M+H, M+Na, M+K	-0.22	11.10	290.8	14.64 ± 0.05	15.37 ± 0.14	5.94E-05	-0.72
Cer 42:2;O2	C42H81NO3	670.6095	M+Na	-2.12	10.95	434.2	4.53 ± 0.27	7.09 ± 0.29	1.27E-05	-2.56
Cer 43:0;O2	C43H87NO3	666.6741	M+H, M+Na	-2.57	11.95	302.1	8.29 ± 0.34	6.64 ± 0.36	5.76E-04	1.65
Cer 44:0;O2	C44H89NO3	680.6907	M+H, M+Na	-1.24	12.17	304.8	8.42 ± 0.43	8.96 ± 0.3	8.39E-02	-0.54
Cer 44:0;O3	C44H89NO4	678.6742	M+H-H2O	-2.40	11.68	301.8	5.62 ± 0.13	6.36 ± 0.25	1.84E-03	-0.75
Cer 44:1;O2	C44H87NO3	716.6303	M+K	-2.06	12.00	301.0	0.99 ± 0.69	4.53 ± 0.59	2.39E-04	-3.54
Cer 44:2;O2	C44H85NO3	698.6416	M+Na	-0.92	11.66	295.4	8.29 ± 0.1	9.28 ± 0.2	9.67E-05	-0.99
HexCer 30:1;O2	C36H69NO8	666.4918	M+H-H2O, M+H, M+Na	0.37	6.15	278.2	13.64 ± 0.11	14.77 ± 0.04	1.26E-06	-1.13
HexCer 36:1;O4	C42H81NO10	777.6192	M+NH4	-0.90	9.45	309.0	9.74 ± 0.03	10.45 ± 0.04	7.56E-08	-0.72
HexCer 40:1;O2	C46H89NO8	806.6475	M+H-H2O, M+Na, M+H	-0.68	10.16	311.5	13.31 ± 0.11	15.52 ± 0.05	2.60E-08	-2.21
HexCer 42:0;O2	C48H95NO8	836.6941	M+Na	-1.09	10.97	323.2	11.3 ± 0.25	11.47 ± 0.18	2.98E-01	-0.17
HexCer 42:1;O2	C48H93NO8	812.6979	M+H-H2O, M+K, M+H	0.57	10.79	320.6	13.64 ± 0.03	15.54 ± 0.08	1.04E-08	-1.89
HexCer 42:1;O3	C48H93NO9	850.6731	M+Na	-1.45	10.60	323.0	13.48 ± 0.17	12.99 ± 0.08	1.94E-03	0.49
HexCer 42:2;O2	C48H91NO8	832.6639	M+H-H2O, M+H, M+Na	0.16	10.18	317.1	12.19 ± 0.03	15.21 ± 0.09	9.35E-10	-3.02

HexCer 42:2;O2	C48H91NO8	810.6798	M+H-H2O, M+H	-2.46	10.33	317.5	10.28 ± 0.19	12.74 ± 0.21	2.33E-06	-2.47
HexCer 42:2;O2	C48H91NO8	832.6638	M+Na	0.14	10.33	314.1	11.36 ± 0.17	14.09 ± 0.34	6.90E-06	-2.72
Hex2Cer 30:1;O2	C42H79NO13	828.5450	M+Na, M+K, M+H, M+H-H2O	0.80	5.73	305.0	12.73 ± 0.3	14.03 ± 0.06	1.53E-04	-1.29
Hex2Cer 36:1;O2	C48H91NO13	890.6567	M+H, M+Na	0.43	8.37	331.6	-1.03 ± 0.5	11.36 ± 0.16	6.02E-09	-12.39
Hex2Cer 40:0;O2	C52H101NO13	970.7166	M+H, M+Na	0.07	10.02	339.7	6.16 ± 0.52	11.15 ± 0.27	2.59E-06	-4.99
Hex2Cer 40:1;O2	C52H99NO13	968.7015	M+Na, M+K	0.70	9.81	336.6	6.97 ± 0.7	13.74 ± 0.13	1.37E-06	-6.77
Hex2Cer 40:1;O2	C52H99NO13	946.7196	M+H-H2O, M+H	0.69	9.81	340.1	7.87 ± 0.09	13.83 ± 0.1	1.86E-10	-5.95
Hex2Cer 42:0;O2	C54H105NO13	976.7653	M+H	-0.56	10.65	349.0	6.05 ± 0.51	10.09 ± 0.25	7.77E-06	-4.04
Hex2Cer 42:1;O2	C54H103NO13	996.7330	M+H, M+Na, M+K, M+H-H2O	0.89	10.44	345.5	10.88 ± 0.1	15.95 ± 0.13	1.14E-09	-5.08
Hex2Cer 42:2;O2	C54H101NO13	972.7349	M+H-H2O, M+H	0.31	9.82	345.9	3.37 ± 1.44	13.66 ± 0.13	7.40E-06	-10.30
Hex2Cer 42:2;O2	C54H101NO13	994.7170	M+Na	0.50	9.82	342.4	0.07 ± 1.36	13.41 ± 0.06	1.16E-06	-13.34
Hex2Cer 42:2;O2	C54H101NO13	994.7166	M+H, M+Na	0.07	10.00	342.4	4.77 ± 0.23	13.08 ± 0.1	8.15E-10	-8.31
Hex3Cer 34:1;O2	C52H97NO18	1046.6600	M+H, M+Na	0.16	7.27	344.8	11.23 ± 0.14	9.72 ± 0.11	2.65E-06	1.51
Hex3Cer 42:1;O2	C60H113NO18	1136.8033	M+H	0.24	10.21	365.7	10.43 ± 0.11	9.35 ± 0.2	7.44E-05	1.09
Hex3Cer 42:1;O2	C60H113NO18	1158.7854	M+Na	0.36	10.21	365.4	9.23 ± 0.17	7.17 ± 0.45	1.42E-04	2.07
Hex3-Cer 44:1;O2	C62H117NO18	1146.8238	M+H-H2O	0.05	7.95	282.4	5.96 ± 0.5	6.31 ± 0.22	2.48E-01	-0.35
Hex3Cer 40:1;O2	C58H109NO18	1130.7518	M+Na	-1.75	9.58	359.4	8.56 ± 0.32	7.05 ± 0.38	9.03E-04	1.50

[a]Property reported for the most abundant ion; [b]If more ions are found, the most abundant is the first one reported. [c]Statistical significance was set at p<0.05 by unpaired two-tailed t-test (n=4 per cell line). Expressed in scientific notation.

Table S6. Identified sphingolipids (Lipid ID, formula, observed m/z, adducts, mass error (ppm), retention time (RT, min), collisional cross section (CCS, $Å^2$), average log_2 intensity ± standard deviation, p-value, and fold change (log_2) between $PC9^{BRAFG469A}$ + PDMP and $PC9^{BRAFG469A}$ cell lines.

Lipid ID	Formula	Observed m/z[a]	Adducts[b]	Mass Error (ppm)[a]	RT (min)[a]	CCS ($Å^2$)[a]	$PC9^{BRAFG469A}$ + PDMP (ave. ± SD)	$PC9^{BRAFG469A}$ (ave. ± SD)	p-value[c]	Fold change (log_2) $PC9^{BRAFG469A}$ + PDMP *vs* $PC9^{BRAFG469A}$
SM 42:2;O2	C47H93N2O6P	835.6663	M+Na	-0.02	10.00	322.5	8.69 ± 0.37	6.85 ± 0.43	6.38E-04	1.84
SM 42:1;O2	C47H95N2O6P	837.6807	M+Na	-1.62	10.06	325.8	8.07 ± 0.33	7.11 ± 0.25	3.78E-03	0.95
SM 42:1;O2	C47H95N2O6P	853.6578	M+K	2.36	10.45	328.8	8.52 ± 0.21	6.51 ± 0.37	7.52E-05	2.01
SM 42:0;O2	C47H97N2O6P	817.7155	M+H, M+Na	-0.23	10.66	329.5	11.79 ± 0.1	11.72 ± 0.4	7.47E-01	0.07
SM 30:1;O2	C35H71N2O6P	647.5129	M+H, M+Na	1.04	5.64	287.2	11.64 ± 0.11	11.6 ± 0.09	6.16E-01	0.04
SM 34:2;O3	C39H77N2O7P	717.5545	M+H	0.51	6.46	301.8	7.53 ± 0.11	6.75 ± 0.1	4.27E-05	0.78
SM 34:2;O2	C39H77N2O6P	701.5600	M+H, M+Na	1.16	6.80	295.6	11.86 ± 0.08	11.08 ± 0.08	8.98E-06	0.78
SM 34:1;O2	C39H79N2O6P	725.5577	M+Na, M+K, M+H-H2O	1.25	7.48	301.6	12.88 ± 0.11	12.61 ± 0.07	5.89E-03	0.27
SM 34:0;O2	C39H81N2O6P	705.5914	M+H, M+Na	1.25	7.80	305.3	13.35 ± 0.07	13.35 ± 0.03	9.66E-01	0
SM 35:1;O2	C40H81N2O6P	717.5905	M+H, M+Na	0.03	7.93	305.1	10.61 ± 0.21	9.85 ± 0.15	1.13E-03	0.76
SM 36:1;O2	C41H83N2O6P	753.5887	M+Na	0.88	8.32	307.6	10.39 ± 0.09	10.04 ± 0.1	1.68E-03	0.35
SM 38:1;O2	C43H87N2O6P	759.6379	M+H, M+Na	0.64	9.10	314.0	12.09 ± 0.2	11.19 ± 0.12	2.83E-04	0.9
SM 40:2;O2	C45H89N2O6P	807.6352	M+Na	0.15	9.32	316.4	8.17 ± 0.07	6.77 ± 0.16	3.74E-06	1.4
SM 42:3;O2	C47H91N2O6P	811.6696	M+Na, M+K, M+H	0.96	9.32	319.6	13.23 ± 0.21	11.89 ± 0.2	9.10E-05	1.34
SM 40:1;O2	C45H91N2O6P	825.6252	M+K	0.73	9.80	319.3	6.66 ± 0.2	4.19 ± 0.36	2.00E-05	2.47
SM 40:1;O2	C45H91N2O6P	809.6514	M+Na	0.89	9.81	319.6	11.17 ± 0.11	10.05 ± 0.13	1.20E-05	1.11

SM 42:2;O2	C47H93N2O6P	835.6671	M+Na, M+K	0.93	9.82	322.5	12.46 ± 0.09	10.85 ± 0.11	5.98E-07	1.61
Cer 42:2;O2	C42H81NO3	630.6176	M+H-H2O, M+H	-1.22	10.16	290.8	8.08 ± 0.37	10.23 ± 0.1	3.12E-05	-2.15
Cer 42:0;O3	C42H85NO4	650.6429	M+H-H2O	-2.54	10.38	296.8	5.76 ± 0.4	8.74 ± 0.04	5.91E-06	-2.98
Cer 42:0;O3	C42H85NO4	650.6432	M+H-H2O	-2.16	10.42	346.7	2.84 ± 0.34	5.51 ± 0.52	1.36E-04	-2.67
Cer 40:0;O2	C40H81NO3	646.6112	M+Na	0.61	10.49	290.4	4.98 ± 0.46	2.19 ± 0.42	1.07E-04	2.79
Cer 42:1;O3	C42H83NO4	648.6277	M+H-H2O	-1.79	10.57	296.8	6.88 ± 0.16	6.46 ± 0.57	2.14E-01	0.41
Cer 42:1;O3	C42H83NO4	666.6395	M+H	0.10	10.57	322.7	3.76 ± 0.19	3.05 ± 0.87	1.65E-01	0.7
Cer 42:2;O	C42H81NO2	614.6230	M+H-H2O	-0.70	10.76	288.0	5.08 ± 0.33	7.91 ± 0.28	1.28E-05	-2.83
Cer 42:1;O2	C42H83NO3	632.6339	M+H-H2O, M+H	-0.07	10.76	294.0	9.59 ± 0.17	11.94 ± 0.25	4.54E-06	-2.35
Cer 40:1;O2	C40H79NO3	604.6029	M+H-H2O, M+Na, M+H	0.23	10.92	288.2	10.63 ± 0.28	10.74 ± 0.34	6.27E-01	-0.11
Cer 42:0;O2	C42H85NO3	652.6607	M+H-H2O, M+H	0.70	10.94	300.0	7.66 ± 0.24	10.75 ± 0.47	2.44E-05	-3.08
Cer 42:2;O2	C42H81NO3	630.6185	M+H-H2O, M+Na, M+H	0.26	11.07	290.8	10.57 ± 0.12	9.79 ± 0.08	2.79E-05	0.79
Cer 40:0;O2	C40H81NO3	606.6179	M+H-H2O	-0.67	11.10	284.9	5.45 ± 0.4	7.36 ± 0.55	1.41E-03	-1.9
Cer 40:0;O2	C40H81NO3	624.6289	M+H, M+Na	-0.01	11.11	290.9	8.42 ± 0.34	10.14 ± 0.58	2.14E-03	-1.73
Cer 42:1;O2	C42H83NO3	650.6445	M+H, M+Na	-0.10	11.13	296.8	10.54 ± 0.17	10.01 ± 0.04	8.26E-04	0.53
Cer 42:1;O2	C42H83NO3	632.6344	M+H-H2O, M+K, M+H, M+Na	0.64	11.47	294.0	12.97 ± 0.14	12.42 ± 0.79	2.16E-01	0.55
Cer 42:2;O	C42H81NO2	614.6230	M+H-H2O	-0.63	11.48	291.2	7.01 ± 0.27	6.28 ± 1.04	2.22E-01	0.73
Cer 42:1;O	C42H83NO2	634.6492	M+H	-0.71	11.64	293.9	7.22 ± 0.26	7.89 ± 0.85	1.83E-01	-0.67
Cer 42:0;O2	C42H85NO3	652.6605	M+H, M+Na	0.49	11.65	296.7	9.62 ± 0.2	10.47 ± 1.13	1.88E-01	-0.85

Cer 42:0;O	C42H85NO2	636.6646	M+H	-1.05	12.02	297.1	3.38 ± 0.54	4.2 ± 0.92	1.73E-01	-0.82
Cer 30:1;O2	C30H59NO3	464.4454	M+H-H2O	-1.58	5.02	257.3	6.27 ± 0.34	6.23 ± 0.32	8.91E-01	0.03
Cer 30:1;O2	C30H59NO3	464.4461	M+H-H2O, M+H	-0.26	5.75	251.2	7.31 ± 0.1	7.27 ± 0.07	5.75E-01	0.04
Cer 30:1;O2	C30H59NO3	464.4465	M+H-H2O, M+H	0.52	6.17	251.2	8.25 ± 0.1	8.22 ± 0.06	6.91E-01	0.03
Cer 30:1;O2	C30H59NO3	464.4466	M+H-H2O, M+K, M+Na	0.80	7.15	251.2	12.04 ± 0.1	12.03 ± 0.04	7.84E-01	0.01
Cer 32:1;O2	C32H63NO3	492.4768	M+H-H2O, M+Na	-1.42	8.03	256.5	7.77 ± 0.08	7.95 ± 0.14	6.67E-02	-0.18
Cer 34:2;O2	C34H65NO3	518.4918	M+H-H2O	-2.58	8.22	258.9	6.42 ± 0.08	5.35 ± 0.15	1.37E-05	1.08
Cer 34:1;O2	C34H67NO3	520.5090	M+H-H2O, M+K, M+Na	0.42	8.85	265.0	12.39 ± 0.14	12.27 ± 0.11	2.24E-01	0.12
Cer 34:0;O2	C34H69NO3	540.5349	M+H-H2O, M+H	-0.36	9.13	267.6	9.66 ± 0.41	11.14 ± 0.13	4.51E-04	-1.48
Cer 35:1;O2	C35H69NO3	534.5235	M+H-H2O	-1.82	9.23	267.8	4.74 ± 0.29	4.89 ± 0.09	3.41E-01	-0.16
Cer 40:1;O2	C40H79NO3	604.6024	M+H-H2O	-0.58	9.55	288.2	1.94 ± 0.44	3.26 ± 0.49	7.11E-03	-1.31
Cer 36:1;O2	C36H71NO3	548.5400	M+H-H2O, M+K, M+Na	-0.21	9.61	273.7	10.44 ± 0.36	10.3 ± 0.1	5.02E-01	0.13
Cer 40:0;O3	C40H81NO4	622.6116	M+H-H2O	-2.71	9.78	340.6	2.08 ± 0.42	5.29 ± 0.31	1.76E-05	-3.2
HexCer 41:2;O2	C47H89NO8	796.6663	M+H	0.27	10.26	319.9	5.08 ± 0.37	5 ± 0.69	8.45E-01	0.08
HexCer 44:2;O2	C50H95NO8	860.6940	M+Na	-1.22	10.73	328.7	4.98 ± 0.62	7.38 ± 0.11	2.70E-04	-2.4
HexCer 42:2;O2	C48H91NO8	832.6644	M+H-H2O, M+Na, M+H	0.78	10.16	319.2	12.88 ± 0.25	14.47 ± 0.06	1.75E-05	-1.59
HexCer 42:2;O2	C48H91NO8	810.6809	M+H	-1.10	10.31	319.6	8.14 ± 0.33	10.13 ± 0.12	2.80E-05	-1.99
HexCer 40:0;O2	C46H91NO8	786.6821	M+H, M+Na	0.37	10.35	320.1	9.23 ± 0.29	12.26 ± 0.3	7.00E-06	-3.04
HexCer 42:1;O3	C48H93NO9	810.6824	M+H-H2O, M+Na, M+H	0.80	10.55	322.9	11.48 ± 0.11	11.24 ± 0.52	4.08E-01	0.24

HexCer 42:1;O2	C48H93NO8	850.6511	M+K	-2.65	10.76	322.2	8.08 ± 0.22	10.06 ± 0.26	2.28E-05	-1.98
HexCer 43:2;O3	C49H93NO9	857.7168	M+NH4	-2.46	11.14	328.7	2.93 ± 0.53	2.94 ± 0.53	9.78E-01	-0.01
HexCer 34:1;O2	C40H77NO8	682.5616	M+H-H2O, M+H	-0.14	6.34	296.1	6.39 ± 0.28	6.8 ± 0.27	7.68E-02	-0.41
HexCer 42:0;O2	C48H95NO8	836.6952	M+Na	0.28	10.94	322.4	8.35 ± 0.21	11.38 ± 0.47	2.24E-05	-3.03
Hex2Cer 36:1;O2	C48H91NO13	912.6387	M+Na	0.44	8.40	327.7	7.96 ± 0.42	10.72 ± 0.24	2.76E-05	-2.76
Hex2Cer 40:0;O2	C52H101NO13	948.7350	M+H, M+Na	0.41	10.00	347.3	10.02 ± 0.3	11.59 ± 0.33	4.17E-04	-1.57
Hex2Cer 44:2;O2	C56H105NO13	1000.7634	M+H	-2.52	10.40	349.9	5.76 ± 0.51	7.91 ± 0.22	2.43E-04	-2.15
Hex2Cer 42:1;O2	C54H103NO13	974.7516	M+H, M+Na, M+H-H2O	1.44	10.41	343.5	13.38 ± 0.08	14.68 ± 0.29	1.24E-04	-1.3
Hex2Cer 42:0;O2	C54H105NO13	976.7664	M+H, M+Na	0.54	10.60	350.3	10.22 ± 0.13	11.69 ± 0.47	8.75E-04	-1.48
Hex2Cer 44:1;O2	C56H107NO13	1002.7824	M+H	0.91	10.97	353.2	6.29 ± 0.2	8.3 ± 0.5	3.10E-04	-2.01
Hex2Cer 30:1;O2	C42H79NO13	806.5635	M+H, M+Na, M+H-H2O	1.37	5.76	303.3	11.47 ± 0.1	11.45 ± 0.09	7.47E-01	0.02
Hex2Cer 32:1;O2	C44H83NO13	834.5951	M+H, M+Na, M+H-H2O	1.63	6.67	312.6	5.73 ± 0.27	7.88 ± 0.12	7.11E-06	-2.14
Hex2Cer 34:1;O2	C46H87NO13	862.6257	M+H, M+Na, M+K, M+H-H2O	0.83	7.55	322.0	11.82 ± 0.07	13.45 ± 0.08	6.64E-08	-1.63
Hex2Cer 36:1;O2	C48H91NO13	890.6566	M+H-H2O, M+H	0.33	8.37	328.1	8.81 ± 0.17	10.95 ± 0.08	3.91E-07	-2.15
Hex2Cer 38:1;O2	C50H95NO13	940.6705	M+H, M+Na, M+H-H2O	0.97	9.11	333.9	8.11 ± 0.23	10.25 ± 0.15	4.12E-06	-2.14
Hex2Cer 40:1;O2	C52H99NO13	946.7200	M+Na, M+K, M+H, M+H-H2O	1.14	9.78	340.6	12.32 ± 0.15	13.68 ± 0.17	2.24E-05	-1.36
Hex2Cer 42:2;O2	C54H101NO13	972.7355	M+H, M+Na, M+H-H2O	0.98	9.80	343.5	12.39 ± 0.15	13.86 ± 0.09	2.41E-06	-1.47
Hex2Cer 42:2;O2	C54H101NO13	972.7337	M+H, M+Na	-0.94	9.98	343.5	6.81 ± 0.66	9.61 ± 0.33	2.66E-04	-2.8

Hex2Cer 43:1;O2	C55H105NO13	988.7666	M+H	0.75	10.70	350.1	4.53 ± 0.63	6.99 ± 0.47	7.67E-04	-2.46
Hex3Cer 42:1;O2	C60H113NO18	1136.8042	M+H, M+Na, M+H-H2O	0.97	10.19	368.3	9.19 ± 0.13	9.25 ± 0.48	8.02E-01	-0.06
Hex3Cer 34:1;O2	C52H97NO18	1024.6796	M+H, M+Na, M+H-H2O	1.65	7.28	342.7	8.26 ± 0.25	8.77 ± 0.23	2.35E-02	-0.51
Hex3Cer 40:1;O2	C58H109NO18	1108.7728	M+H-H2O, M+Na, M+H	0.90	9.55	361.8	7.72 ± 0.42	8.2 ± 0.35	1.25E-01	-0.49
Hex3Cer 42:2;O2	C60H111NO18	1134.7881	M+H, M+Na, M+H-H2O	0.58	9.57	364.9	8.72 ± 0.42	9.25 ± 0.35	1.01E-01	-0.53

[a]Property reported for the most abundant ion; [b]If more ions are found, the most abundant is the first one reported. [c]Statistical significance was set at p<0.05 by unpaired two-tailed t-test (n=4 per cell line).

Table S7. Identified sphingolipids (Lipid ID, formula, observed m/z, adducts, mass error (ppm), retention time (RT, min), collisional cross section (CCS, Å^2), average log_2 intensity ± standard deviation, p-value, and fold change (log_2) between PC9T790M^{clA} + PDMP and PC9T790M^{clA} cell lines.

Lipid ID	Formula	Observed m/z[a]	Adducts[b]	Mass Error (ppm)[a]	RT (min)[a]	CCS (Å^2)[a]	PC9T790M^{clA} + PDMP (ave. ± SD)	PC9T790M^{clA} (ave. ± SD)	p-value[c]	Fold change (log_2) PC9T790M^{clA} + PDMP *vs* PC9T790M^{clA}
SM 32:2;O2	C37H73N2O6P	673.5271	M+H	-1.25	5.78	287.9	5.61 ± 0.25	5.14 ± 0.71	2.58E-01	0.47
SM 32:1;O2	C37H75N2O6P	675.5441	M+H, M+Na	0.90	6.54	291.0	11.97 ± 0.16	12.06 ± 0.3	6.34E-01	-0.09
SM 34:1;O2	C39H79N2O6P	725.5576	M+Na, M+K	1.14	7.46	299.3	13.83 ± 0.26	13.77 ± 0.18	7.28E-01	0.06
SM 34:0;O2	C39H81N2O6P	705.5911	M+H	0.74	7.79	302.9	13.03 ± 0.17	13.6 ± 0.31	1.81E-02	-0.57
SM 40:2;O2	C45H89N2O6P	807.6339	M+Na	-1.46	9.29	313.5	6.7 ± 0.24	6.61 ± 0.45	7.35E-01	0.09
SM 42:3;O2	C47H91N2O6P	811.6690	M+H, M+Na	0.28	9.30	316.6	12.99 ± 0.15	12.15 ± 0.56	2.66E-02	0.84
SM 40:1;O2	C45H91N2O6P	787.6691	M+H	0.43	9.82	317.1	14.09 ± 0.19	13.52 ± 0.43	5.12E-02	0.57
SM 42:2;O2	C47H93N2O6P	813.6851	M+H	0.91	9.82	323.0	15.53 ± 0.18	14.83 ± 0.3	7.36E-03	0.70
SM 40:1;O2	C45H91N2O6P	809.6506	M+Na	-0.19	9.83	319.9	9.78 ± 0.39	8.46 ± 1.1	6.40E-02	1.33
SM 42:2;O2	C47H93N2O6P	813.6850	M+H, M+Na	0.75	10.00	319.8	13.45 ± 0.15	12.8 ± 0.31	9.10E-03	0.65
SM 42:1;O2	C47H95N2O6P	837.6822	M+Na, M+K	0.30	10.45	322.5	12.12 ± 0.22	11.12 ± 0.57	1.69E-02	1.00
SM 42:0;O2	C47H97N2O6P	817.7157	M+H, M+Na	-0.03	10.66	326.1	12.05 ± 0.24	12.11 ± 0.41	8.19E-01	-0.06
SM 44:1;O2	C49H99N2O6P	843.7304	M+H	-1.09	11.04	328.8	8.68 ± 0.24	6.35 ± 1.18	8.34E-03	2.33
Cer 34:2;O2	C34H65NO3	518.4918	M+H-H2O, M+Na	-2.64	8.15	260.7	6.08 ± 0.35	5.85 ± 0.12	2.68E-01	0.23
Cer 34:0;O2	C34H69NO3	562.5170	M+Na	-0.07	9.06	265.7	7.97 ± 0.23	9.21 ± 0.39	1.47E-03	-1.24
Cer 36:1;O2	C36H71NO3	588.5330	M+H-H2O, M+Na	0.69	9.55	271.2	11.06 ± 0.26	10.62 ± 0.45	1.40E-01	0.44

Cer 42:1;O2	C42H83NO3	632.6327	M+H-H2O, M+H	-2.00	10.73	295.0	7.25 ± 0.46	10.1 ± 0.5	1.57E-04	-2.86
Cer 40:1;O2	C40H79NO3	604.6024	M+H-H2O	-0.53	10.86	289.4	10.31 ± 0.36	9.86 ± 0.45	1.61E-01	0.46
Cer 42:2;O2	C42H81NO3	630.6185	M+H-H2O, M+H, M+Na	0.16	10.88	292.0	13.38 ± 0.22	12.11 ± 0.28	3.77E-04	1.27
Cer 48:2;O2	C48H93NO3	749.7489	M+NH4	-0.67	11.01	321.0	5.91 ± 0.56	4.27 ± 1.05	3.31E-02	1.64
Cer 42:0;O3	C42H85NO4	650.6436	M+H-H2O	-1.42	11.08	294.6	9.25 ± 0.23	8.67 ± 0.57	1.09E-01	0.58
Cer 41:1;O2	C41H81NO3	618.6174	M+H-H2O, M+Na	-1.55	11.16	292.2	8.56 ± 0.49	7.15 ± 0.53	7.83E-03	1.41
Cer 42:1;O2	C42H83NO3	632.6341	M+H-H2O, M+H, M+K, M+Na	0.21	11.43	295.0	13.11 ± 0.37	12.21 ± 0.42	1.81E-02	0.90
HexCer 40:1;O2	C46H89NO8	806.6474	M+H-H2O, M+Na, M+H	-0.83	10.12	313.5	10.64 ± 0.29	13.21 ± 0.47	8.76E-05	-2.57
HexCer 42:2;O2	C48H91NO8	832.6638	M+NH4, M+Na, M+K, M+H, M+H-H2O	0.07	10.13	316.2	12.06 ± 0.11	13.76 ± 0.37	1.15E-04	-1.70
HexCer 42:2;O2	C48H91NO8	832.6635	M+H-H2O, M+Na, M+H	-0.25	10.29	316.2	9.49 ± 0.2	10.96 ± 0.52	1.82E-03	-1.48
HexCer 41:1;O2	C47H91NO8	820.6633	M+H, M+Na, M+H-H2O	-0.51	10.43	316.5	6.75 ± 0.46	10.37 ± 0.67	1.09E-04	-3.62
HexCer 42:1;O2	C48H93NO8	812.6978	M+H, M+K, M+Na, M+H-H2O	0.47	10.74	319.8	12.59 ± 0.24	14.53 ± 0.4	1.56E-04	-1.95
HexCer 42:0;O2	C48H95NO8	814.7120	M+H, M+Na	-1.25	10.92	323.0	8.1 ± 0.54	10.55 ± 0.6	9.25E-04	-2.45
Hex2Cer 34:1;O2	C46H87NO13	862.6254	M+H-H2O, M+H	0.44	7.52	318.9	12.09 ± 0.17	13.55 ± 0.21	3.60E-05	-1.46
Hex2Cer 36:1;O2	C48H91NO13	890.6561	M+H-H2O, M+H	-0.29	8.35	324.8	9.18 ± 0.22	11.37 ± 0.26	1.35E-05	-2.19
Hex2Cer 32:0;O2	C44H85NO13	818.5984	M+H-H2O	-0.48	8.93	310.2	12.29 ± 0.14	12.52 ± 0.29	2.02E-01	-0.23

Hex2Cer 40:1;O2	C52H99NO13	946.7191	M+H	0.13	9.80	336.7	11.76 ± 0.48	13.06 ± 0.38	5.27E-03	-1.30
Hex2Cer 42:2;O2	C54H101NO13	972.7346	M+H	0.04	9.82	339.5	11.34 ± 0.36	13.35 ± 0.28	1.21E-04	-2.01
Hex2Cer 42:2;O2	C54H101NO13	972.7338	M+H	-0.84	9.96	339.5	9.05 ± 0.08	11.09 ± 0.41	6.49E-05	-2.03
Hex2Cer 40:0;O2	C52H101NO13	948.7351	M+H, M+Na	0.53	10.00	343.1	10.78 ± 0.3	11.53 ± 0.54	5.15E-02	-0.75
Hex2Cer 42:0;O2	C54H105NO13	976.7663	M+H, M+Na	0.45	10.61	345.9	10.55 ± 0.38	11.54 ± 0.53	2.40E-02	-0.99
Hex2Cer 36:1;O2	C48H91NO13	912.6380	M+Na	-0.30	8.37	327.6	9.24 ± 0.25	11.78 ± 0.09	1.36E-06	-2.55
Hex3Cer 34:1;O2	C52H97NO18	1024.6784	M+H, M+Na	0.51	7.27	338.7	6.8 ± 0.26	6.83 ± 0.77	9.51E-01	-0.03
Hex3Cer 34:1;O2	C52H97NO18	1006.6674	M+H-H2O	0.07	7.29	335.7	-1.48 ± 3.42	-1.35 ± 2.95	9.56E-01	-0.13
Hex3Cer 40:1;O2	C58H109NO18	1108.7719	M+H, M+Na	0.06	9.56	356.9	5.09 ± 0.26	4.35 ± 1.04	2.14E-01	0.75
Hex3Cer 42:2;O2	C60H111NO18	1134.7876	M+H-H2O, M+H	0.13	9.57	359.8	5.96 ± 0.48	6.09 ± 1.04	8.35E-01	-0.12
Hex3Cer 42:2;O2	C60H111NO18	1156.7691	M+Na	-0.21	9.57	359.5	2.36 ± 1.21	2.41 ± 0.82	9.50E-01	-0.05
Hex3Cer 42:1;O2	C60H113NO18	1136.8034	M+H-H2O, M+H, M+Na	0.33	10.19	363.1	8.07 ± 0.41	7.6 ± 0.81	3.41E-01	0.47

[a]Property reported for the most abundant ion; [b]If more ions are found, the most abundant is the first one reported. [c]Statistical significance was set at $p<0.05$ by unpaired two-tailed t-test (n=4 per cell line). Expressed in scientific notation.

Table S8. Identified sphingolipids (lipid ID, formula, observed m/z, adducts, mass error (ppm), retention time (RT, min), collisional cross section (CCS, Å^2), average log_2 intensity ± standard deviation, p-value, and fold change (log_2) between PC9T790M^{clC} + PDMP and PC9T790M^{clC} cell lines.

Lipid ID	Formula	Observed m/z[a]	Adducts[b]	Mass Error (ppm)[a]	RT (min)[a]	CCS (Å^2)[a]	PC9T790M^{clC} + PDMP (ave. ± SD)	PC9T790M^{clC} (ave. ± SD)	p-value[c]	Fold change (log_2) PC9T790M^{clC} + PDMP *vs* PC9T790M
SM 32:2;O2	C37H73N2O6P	673.5271	M+H	-1.25	5.78	287.9	6.99 ± 0.28	6.54 ± 0.97	4.10E-01	0.45
SM 32:1;O2	C37H75N2O6P	675.5441	M+H, M+Na	0.90	6.54	291.0	12.24 ± 0.22	12.01 ± 0.42	3.77E-01	0.23
SM 34:1;O2	C39H79N2O6P	725.5576	M+Na, M+K	1.14	7.46	299.3	13.8 ± 0.3	13.72 ± 0.29	6.95E-01	0.09
SM 34:0;O2	C39H81N2O6P	705.5911	M+H	0.74	7.79	302.9	14.71 ± 0.28	14.63 ± 0.56	8.02E-01	0.08
SM 40:2;O2	C45H89N2O6P	807.6339	M+Na	-1.46	9.29	313.5	9.45 ± 0.24	8.46 ± 0.96	9.09E-02	1.00
SM 42:3;O2	C47H91N2O6P	811.6690	M+H, M+Na	0.28	9.30	316.6	14.51 ± 0.19	13.68 ± 0.6	3.82E-02	0.83
SM 40:1;O2	C45H91N2O6P	787.6691	M+H	0.43	9.82	317.1	14.93 ± 0.22	14.01 ± 0.41	7.62E-03	0.92
SM 42:2;O2	C47H93N2O6P	813.6851	M+H	0.91	9.82	323.0	15.89 ± 0.19	14.86 ± 0.4	3.44E-03	1.02
SM 40:1;O2	C45H91N2O6P	809.6506	M+Na	-0.19	9.83	319.9	11.39 ± 0.44	8.96 ± 1.63	2.77E-02	2.44
SM 42:2;O2	C47H93N2O6P	813.6850	M+H, M+Na	0.75	10.00	319.8	15.1 ± 0.22	14.58 ± 0.37	5.10E-02	0.52
SM 42:1;O2	C47H95N2O6P	837.6822	M+Na, M+K	0.30	10.45	322.5	13.23 ± 0.36	11.69 ± 0.66	6.36E-03	1.54
SM 42:0;O2	C47H97N2O6P	817.7157	M+H, M+Na	-0.03	10.66	326.1	13.27 ± 0.32	12.83 ± 0.33	9.97E-02	0.45
SM 44:1;O2	C49H99N2O6P	843.7304	M+H	-1.09	11.04	328.8	10.17 ± 0.35	7.94 ± 1.1	8.47E-03	2.23
Cer 34:2;O2	C34H65NO3	518.4918	M+H-H2O, M+Na	-2.64	8.15	260.7	9.09 ± 0.36	9.54 ± 0.33	1.22E-01	-0.44
Cer 34:0;O2	C34H69NO3	562.5170	M+Na	-0.07	9.06	265.7	8.24 ± 0.36	8.84 ± 0.23	3.09E-02	-0.60
Cer 36:1;O2	C36H71NO3	588.5330	M+H-H2O, M+Na	0.69	9.55	271.2	10.34 ± 0.4	9.38 ± 0.58	3.32E-02	0.96

Cer 42:1;O2	C42H83NO3	632.6327	M+H-H2O, M+H	-2.00	10.73	295.0	5.87 ± 0.99	9.35 ± 0.67	1.12E-03	-3.48
Cer 40:1;O2	C40H79NO3	604.6024	M+H-H2O	-0.53	10.86	289.4	10.58 ± 0.42	9.39 ± 0.46	9.11E-03	1.19
Cer 42:2;O2	C42H81NO3	630.6185	M+H-H2O, M+H, M+Na	0.16	10.88	292.0	13.13 ± 0.26	11.39 ± 0.49	8.06E-04	1.74
Cer 48:2;O2	C48H93NO3	749.7489	M+NH4	-0.67	11.01	321.0	8.12 ± 0.35	7.71 ± 0.73	3.47E-01	0.41
Cer 42:0;O3	C42H85NO4	650.6436	M+H-H2O	-1.42	11.08	294.6	9.6 ± 0.37	8.57 ± 0.79	5.73E-02	1.03
Cer 41:1;O2	C41H81NO3	618.6174	M+H-H2O, M+Na	-1.55	11.16	292.2	9.32 ± 0.52	7.04 ± 1.04	7.96E-03	2.27
Cer 42:1;O2	C42H83NO3	632.6341	M+H-H2O, M+H, M+K, M+Na	0.21	11.43	295.0	13.49 ± 0.44	12.09 ± 0.28	1.60E-03	1.41
HexCer 40:1;O2	C46H89NO8	806.6474	M+H-H2O, M+Na, M+H	-0.83	10.12	313.5	9.66 ± 0.63	12.23 ± 0.64	1.26E-03	-2.56
HexCer 42:2;O2	C48H91NO8	832.6638	M+NH4, M+Na, M+K, M+H, M+H-H2O	0.07	10.13	316.2	11.55 ± 0.34	12.7 ± 0.61	1.60E-02	-1.15
HexCer 42:2;O2	C48H91NO8	832.6635	M+H-H2O, M+Na, M+H	-0.25	10.29	316.2	10.58 ± 0.43	12.24 ± 0.57	3.55E-03	-1.65
HexCer 41:1;O2	C47H91NO8	820.6633	M+H, M+Na, M+H-H2O	-0.51	10.43	316.5	5.45 ± 0.92	8.97 ± 1.44	6.15E-03	-3.53
HexCer 42:1;O2	C48H93NO8	812.6978	M+H, M+K, M+Na, M+H-H2O	0.47	10.74	319.8	11.96 ± 0.45	13.96 ± 0.31	3.30E-04	-2.00
HexCer 42:0;O2	C48H95NO8	814.7120	M+H, M+Na	-1.25	10.92	323.0	7.11 ± 0.89	9.7 ± 0.6	2.96E-03	-2.59
Hex2Cer 34:1;O2	C46H87NO13	862.6254	M+H-H2O, M+H	0.44	7.52	318.9	10.68 ± 0.13	11.94 ± 0.23	7.18E-05	-1.26
Hex2Cer 36:1;O2	C48H91NO13	890.6561	M+H-H2O, M+H	-0.29	8.35	324.8	5.25 ± 0.6	7.46 ± 0.24	4.89E-04	-2.20
Hex2Cer 32:0;O2	C44H85NO13	818.5984	M+H-H2O	-0.48	8.93	310.2	12.75 ± 0.2	12.77 ± 0.4	9.09E-01	-0.03

Hex2Cer 40:1;O2	C52H99NO13	946.7191	M+H	0.13	9.80	336.7	10.1 ± 0.63	11.07 ± 0.39	3.98E-02	-0.97
Hex2Cer 42:2;O2	C54H101NO13	972.7346	M+H	0.04	9.82	339.5	9.2 ± 0.43	11.13 ± 0.52	1.27E-03	-1.93
Hex2Cer 42:2;O2	C54H101NO13	972.7338	M+H	-0.84	9.96	339.5	9.97 ± 0.3	11.17 ± 0.49	6.10E-03	-1.20
Hex2Cer 40:0;O2	C52H101NO13	948.7351	M+H, M+Na	0.53	10.00	343.1	9.59 ± 0.58	9.84 ± 0.7	6.04E-01	-0.25
Hex2Cer 42:0;O2	C54H105NO13	976.7663	M+H, M+Na	0.45	10.61	345.9	9.78 ± 0.58	10.24 ± 0.34	2.20E-01	-0.46
Hex2Cer 36:1;O2	C48H91NO13	912.6380	M+Na	-0.30	8.37	327.6	1.34 ± 2	7.32 ± 0.7	1.32E-03	-5.98
Hex3Cer 34:1;O2	C52H97NO18	1024.6784	M+H, M+Na	0.51	7.27	338.7	10.9 ± 0.44	11.86 ± 0.56	3.53E-02	-0.96
Hex3Cer 34:1;O2	C52H97NO18	1006.6674	M+H-H2O	0.07	7.29	335.7	6.91 ± 0.53	8.32 ± 0.73	2.01E-02	-1.42
Hex3Cer 40:1;O2	C58H109NO18	1108.7719	M+H, M+Na	0.06	9.56	356.9	11.07 ± 0.39	11.61 ± 0.49	1.31E-01	-0.54
Hex3Cer 42:2;O2	C60H111NO18	1134.7876	M+H-H2O, M+H	0.13	9.57	359.8	10.2 ± 0.42	11.54 ± 0.4	3.58E-03	-1.34
Hex3Cer 42:2;O2	C60H111NO18	1156.7691	M+Na	-0.21	9.57	359.5	8.66 ± 0.8	9.69 ± 1.7	3.19E-01	-1.02
Hex3Cer 42:1;O2	C60H113NO18	1136.8034	M+H-H2O, M+H, M+Na	0.33	10.19	363.1	12.7 ± 0.37	13.4 ± 0.25	2.05E-02	-0.70

[a]Property reported for the most abundant ion; [b]If more ions are found, the most abundant is the first one reported. [c]Statistical significance was set at p<0.05 by unpaired two-tailed t-test (n=4 per cell line). Expressed in scientific notation.

Table S9. Identified sphingolipids (Lipid ID, formula, observed m/z, adducts, mass error (ppm), retention time (RT, min), collisional cross section (CCS, Å^2), average log_2 intensity ± standard deviation, p-value, and fold change (log_2) between PC9T790M^{C797S} + PDMP and PC9T790M^{C797S} cell lines.

Lipid ID	Formula	Observed m/z[a]	Adducts[b]	Mass Error (ppm)[a]	RT (min)[a]	CCS (Å^2)[a]	PC9T790M^{C797S} + PDMP (ave. ± SD)	PC9T790M^{C797S} (ave. ± SD)	p-value[c]	Fold change (log_2) PC9T790M^{C797S} + PDMP *vs* PC9T790M^{C797S}
SM 30:1;O2	C35H71N2O6P	647.5124	M+H, M+K, M+Na	0.21	5.63	284.5	13.99 ± 0.12	13.73 ± 0.19	6.46E-02	0.26
SM 32:0;O2	C37H77N2O6P	677.5592	M+H, M+Na	0.04	6.95	298.8	12.12 ± 0.13	12.17 ± 0.16	6.28E-01	-0.05
SM 32:1;O2	C37H75N2O6P	675.5439	M+Na, M+K, M+H	0.57	6.59	292.8	14.82 ± 0.13	14.57 ± 0.19	6.87E-02	0.25
SM 32:2;O2	C37H73N2O6P	673.5276	M+H, M+Na	-0.44	5.83	286.9	10.5 ± 0.08	10.4 ± 0.19	3.64E-01	0.10
SM 34:1;O2	C39H79N2O6P	725.5569	M+Na	0.17	7.46	300.9	12.17 ± 0.38	12.27 ± 0.52	7.63E-01	-0.10
SM 34:2;O2	C39H77N2O6P	739.5146	M+K	-0.72	6.82	297.6	8.7 ± 0.05	8.59 ± 0.12	1.24E-01	0.11
SM 34:2;O2	C39H77N2O6P	723.5424	M+Na	1.75	7.03	297.9	4.71 ± 0.39	4.13 ± 0.34	6.95E-02	0.57
SM 35:1;O2	C40H81N2O6P	717.5902	M+H, M+Na	-0.46	7.94	304.1	11.71 ± 0.05	11.64 ± 0.08	2.05E-01	0.07
SM 40:1;O2	C45H91N2O6P	809.6511	M+Na	0.51	9.86	320.6	12.63 ± 0.16	11.93 ± 0.17	9.71E-04	0.70
SM 41:1;O2	C46H93N2O6P	801.6844	M+H, M+Na	0.03	10.22	323.9	14.67 ± 0.22	13.72 ± 0.11	2.36E-04	0.95
SM 42:0;O2	C47H97N2O6P	817.7151	M+H, M+Na	-0.74	10.76	329.8	14.7 ± 0.24	14.05 ± 0.09	2.40E-03	0.66
SM 42:1;O2	C47H95N2O6P	853.6581	M+K	2.72	10.55	326.0	10.33 ± 0.04	9.64 ± 0.03	1.92E-07	0.69
SM 42:2;O2	C47H93N2O6P	851.6398	M+K	-0.55	9.88	323.0	6.76 ± 0.46	4.99 ± 0.3	6.35E-04	1.77
SM 42:2;O2	C47H93N2O6P	835.6664	M+Na	0.04	9.88	323.3	12.93 ± 0.19	11.9 ± 0.11	9.87E-05	1.03
SM 42:2;O2	C47H93N2O6P	835.6667	M+Na	0.40	10.08	320.2	13.68 ± 0.03	13.12 ± 0.05	1.67E-06	0.55
SM 44:1;O2	C49H99N2O6P	843.7309	M+H, M+Na	-0.54	11.15	332.4	11.48 ± 0.26	9.62 ± 0.11	1.30E-05	1.86

Cer 30:1;O2	C30H59NO3	464.4452	M+H-H2O, M+H	-2.17	5.74	251.1	6.98 ± 0.26	6.5 ± 0.5	1.35E-01	0.49
Cer 30:1;O2	C30H59NO3	464.4458	M+H-H2O, M+H	-0.88	6.15	251.1	8.96 ± 0.14	8.63 ± 0.25	5.86E-02	0.33
Cer 30:1;O2	C30H59NO3	464.4460	M+Na, M+K, M+H, M+H-H2O	-0.38	7.13	251.1	13.58 ± 0.17	13.39 ± 0.16	1.40E-01	0.20
Cer 36:1;O2	C36H71NO3	548.5396	M+H-H2O, M+Na, M+H, M+K	-0.91	9.63	272.0	12.09 ± 0.18	11.69 ± 0.24	3.64E-02	0.40
Cer 40:0;O2	C40H81NO3	624.6282	M+H, M+Na	-1.21	11.15	291.0	10.93 ± 0.14	10.96 ± 0.24	8.22E-01	-0.03
Cer 40:1;O2	C40H79NO3	604.6011	M+H-H2O, M+H	-2.59	10.18	288.4	2.99 ± 0.48	8.2 ± 0.06	6.42E-07	-5.21
Cer 40:1;O2	C40H79NO3	604.6023	M+Na, M+K, M+H, M+H-H2O	-0.65	10.93	288.4	13.06 ± 0.08	12.69 ± 0.08	7.42E-04	0.37
Cer 40:2;O2	C40H77NO3	602.5865	M+H-H2O, M+Na, M+H	-0.94	10.49	282.5	11.47 ± 0.05	11.68 ± 0.12	1.40E-02	-0.22
Cer 41:1;O2	C41H81NO3	658.6100	M+Na	-1.34	11.25	287.2	11.41 ± 0.08	11.13 ± 0.12	7.85E-03	0.28
Cer 42:0;O	C42H85NO2	636.6645	M+H, M+Na	-1.26	12.05	296.7	9.72 ± 0.07	10.09 ± 0.09	5.51E-04	-0.38
Cer 42:0;O2	C42H85NO3	652.6601	M+H, M+Na, M+K	-0.26	11.69	299.4	13.72 ± 0.24	13.42 ± 0.25	1.37E-01	0.30
Cer 42:0;O3	C42H85NO4	650.6431	M+H-H2O	-2.22	10.77	320.8	4.22 ± 0.18	8.31 ± 0.05	1.01E-08	-4.08
Cer 42:0;O3	C42H85NO4	650.6438	M+H-H2O	-1.26	11.16	293.4	12.01 ± 0.19	10.9 ± 0.12	5.80E-05	1.11
Cer 42:1;O	C42H83NO2	656.6309	M+Na	-1.07	11.66	284.3	8.43 ± 0.14	9.31 ± 0.1	4.86E-05	-0.88
Cer 42:1;O	C42H83NO2	634.6491	M+H-H2O, M+H	-0.84	11.68	290.7	11.51 ± 0.04	12.01 ± 0.06	8.83E-06	-0.50
Cer 42:1;O2	C42H83NO3	650.6440	M+H	-0.83	11.29	293.4	11.24 ± 0.16	10.94 ± 0.11	2.05E-02	0.30
Cer 42:1;O2	C42H83NO3	672.6263	M+Na, M+K, M+H	-0.23	11.51	289.9	14.65 ± 0.08	14.19 ± 0.11	5.94E-04	0.46
Cer 42:2;O	C42H81NO2	614.6227	M+H-H2O	-1.20	11.51	288.2	9.67 ± 0.22	8.97 ± 0.11	1.21E-03	0.71

Cer 42:2;O	C42H81NO2	632.6332	M+H-H2O, M+H	-1.28	10.77	293.8	6.72 ± 0.39	10.11 ± 0.07	2.49E-06	-3.39
Cer 42:2;O2	C42H81NO3	648.6276	M+H-H2O, M+H	-2.01	10.59	296.4	9.32 ± 0.22	10.18 ± 0.1	3.46E-04	-0.86
Cer 42:2;O2	C42H81NO3	630.6182	M+Na, M+K, M+H, M+H-H2O	-0.30	10.96	293.8	14.32 ± 0.1	13.03 ± 0.06	6.58E-07	1.29
Cer 42:2;O2	C42H81NO3	630.6182	M+H-H2O, M+H, M+Na, M+K	-0.22	11.10	290.8	14.73 ± 0.07	14.64 ± 0.05	9.50E-02	0.09
Cer 42:2;O2	C42H81NO3	670.6095	M+Na	-2.12	10.95	434.2	6.7 ± 0.21	4.53 ± 0.27	1.42E-05	2.18
Cer 43:0;O2	C43H87NO3	666.6741	M+H, M+Na	-2.57	11.95	302.1	8.26 ± 0.43	8.29 ± 0.34	9.05E-01	-0.03
Cer 44:0;O2	C44H89NO3	680.6907	M+H, M+Na	-1.24	12.17	304.8	8.79 ± 0.46	8.42 ± 0.43	2.84E-01	0.37
Cer 44:0;O3	C44H89NO4	678.6742	M+H-H2O	-2.40	11.68	301.8	7.5 ± 0.29	5.62 ± 0.13	2.32E-05	1.88
Cer 44:1;O2	C44H87NO3	716.6303	M+K	-2.06	12.00	301.0	3.85 ± 0.42	0.99 ± 0.69	4.10E-04	2.85
Cer 44:2;O2	C44H85NO3	698.6416	M+Na	-0.92	11.66	295.4	8.97 ± 0.13	8.29 ± 0.1	1.71E-04	0.68
HexCer 30:1;O2	C36H69NO8	666.4918	M+H-H2O, M+H, M+Na	0.37	6.15	278.2	13.88 ± 0.06	13.64 ± 0.11	9.60E-03	0.24
HexCer 36:1;O4	C42H81NO10	777.6192	M+NH4	-0.90	9.45	309.0	9.93 ± 0.07	9.74 ± 0.03	1.81E-03	0.20
HexCer 40:1;O2	C46H89NO8	806.6475	M+H-H2O, M+Na, M+H	-0.68	10.16	311.5	10.91 ± 0.04	13.31 ± 0.11	1.43E-08	-2.40
HexCer 42:0;O2	C48H95NO8	836.6941	M+Na	-1.09	10.97	323.2	8.03 ± 0.31	11.3 ± 0.25	3.20E-06	-3.27
HexCer 42:1;O2	C48H93NO8	812.6979	M+H-H2O, M+K, M+H	0.57	10.79	320.6	11.34 ± 0.19	13.64 ± 0.03	3.25E-07	-2.31
HexCer 42:1;O3	C48H93NO9	850.6731	M+Na	-1.45	10.60	323.0	12.76 ± 0.06	13.48 ± 0.17	2.27E-04	-0.72
HexCer 42:2;O2	C48H91NO8	832.6639	M+H-H2O, M+H, M+Na	0.16	10.18	317.1	11.34 ± 0.08	12.19 ± 0.03	7.32E-07	-0.84
HexCer 42:2;O2	C48H91NO8	810.6798	M+H-H2O, M+H	-2.46	10.33	317.5	4.79 ± 0.67	10.28 ± 0.19	3.96E-06	-5.49

HexCer 42:2;O2	C48H91NO8	832.6638	M+Na	0.14	10.33	314.1	8.16 ± 0.4	11.36 ± 0.17	5.81E-06	-3.20
Hex2Cer 30:1;O2	C42H79NO13	828.5450	M+Na, M+K, M+H, M+H-H2O	0.80	5.73	305.0	13.1 ± 0.14	12.73 ± 0.3	6.93E-02	0.37
Hex2Cer 36:1;O2	C48H91NO13	890.6567	M+H, M+Na	0.43	8.37	331.6	-0.03 ± 0.89	-1.03 ± 0.5	9.71E-02	1.00
Hex2Cer 40:0;O2	C52H101NO13	970.7166	M+H, M+Na	0.07	10.02	339.7	3.38 ± 0.66	6.16 ± 0.52	5.63E-04	-2.78
Hex2Cer 40:1;O2	C52H99NO13	968.7015	M+Na, M+K	0.70	9.81	336.6	6.25 ± 0.69	6.97 ± 0.7	1.91E-01	-0.72
Hex2Cer 40:1;O2	C52H99NO13	946.7196	M+H-H2O, M+H	0.69	9.81	340.1	7.05 ± 0.24	7.87 ± 0.09	7.21E-04	-0.83
Hex2Cer 42:0;O2	C54H105NO13	976.7653	M+H	-0.56	10.65	349.0	2.5 ± 1.01	6.05 ± 0.51	7.46E-04	-3.56
Hex2Cer 42:1;O2	C54H103NO13	996.7330	M+H, M+Na, M+K, M+H-H2O	0.89	10.44	345.5	10.08 ± 0.35	10.88 ± 0.1	4.85E-03	-0.79
Hex2Cer 42:2;O2	C54H101NO13	972.7349	M+H-H2O, M+H	0.31	9.82	345.9	5.86 ± 0.51	3.37 ± 1.44	1.69E-02	2.50
Hex2Cer 42:2;O2	C54H101NO13	994.7170	M+Na	0.50	9.82	342.4	3.84 ± 0.75	0.07 ± 1.36	2.86E-03	3.77
Hex2Cer 42:2;O2	C54H101NO13	994.7166	M+H, M+Na	0.07	10.00	342.4	3.42 ± 0.75	4.77 ± 0.23	1.36E-02	-1.35
Hex3Cer 34:1;O2	C52H97NO18	1046.6600	M+H, M+Na	0.16	7.27	344.8	9.53 ± 0.03	11.23 ± 0.14	2.93E-07	-1.70
Hex3Cer 42:1;O2	C60H113NO18	1136.8033	M+H	0.24	10.21	365.7	9.25 ± 0.28	10.43 ± 0.11	2.24E-04	-1.19
Hex3Cer 42:1;O2	C60H113NO18	1158.7854	M+Na	0.36	10.21	365.4	8.48 ± 0.41	9.23 ± 0.17	1.42E-02	-0.76
Hex3Cer 44:1;O2	C62H117NO18	1146.8238	M+H-H2O	0.05	7.95	282.4	7.26 ± 0.43	5.96 ± 0.5	7.96E-03	1.29
Hex3Cer 40:1;O2	C58H109NO18	1130.7518	M+Na	-1.75	9.58	359.4	6.96 ± 0.42	8.56 ± 0.32	9.23E-04	-1.60

[a]Property reported for the most abundant ion; [b]If more ions are found, the most abundant is the first one reported. [c]Statistical significance was set at $p<0.05$ by unpaired two-tailed t-test (n=4 per cell line). Expressed in scientific notation.

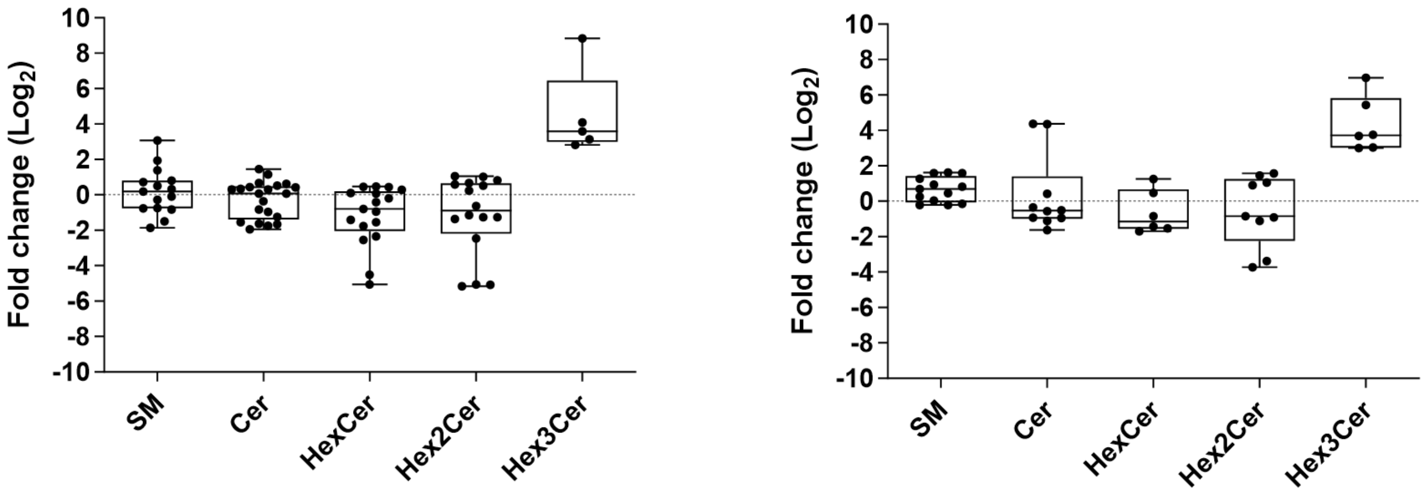

Figure S1. Box and whiskers plot representing fold change (log_2 scale) of putatively identified sphingolipids between $PC9^{T790MclC}$ and $PC9^{T790M}$ performed in two different days (inter-day reproducibility). (A) $PC9^{T790MclC}$ vs $PC9^{T790M}$ (first analysis); (B) $PC9^{T790MclC}$ vs $PC9^{T790M}$ (second analysis). Boxes are determined by the 25th and 75th percentiles and whiskers are determined by minimum and maximum values.

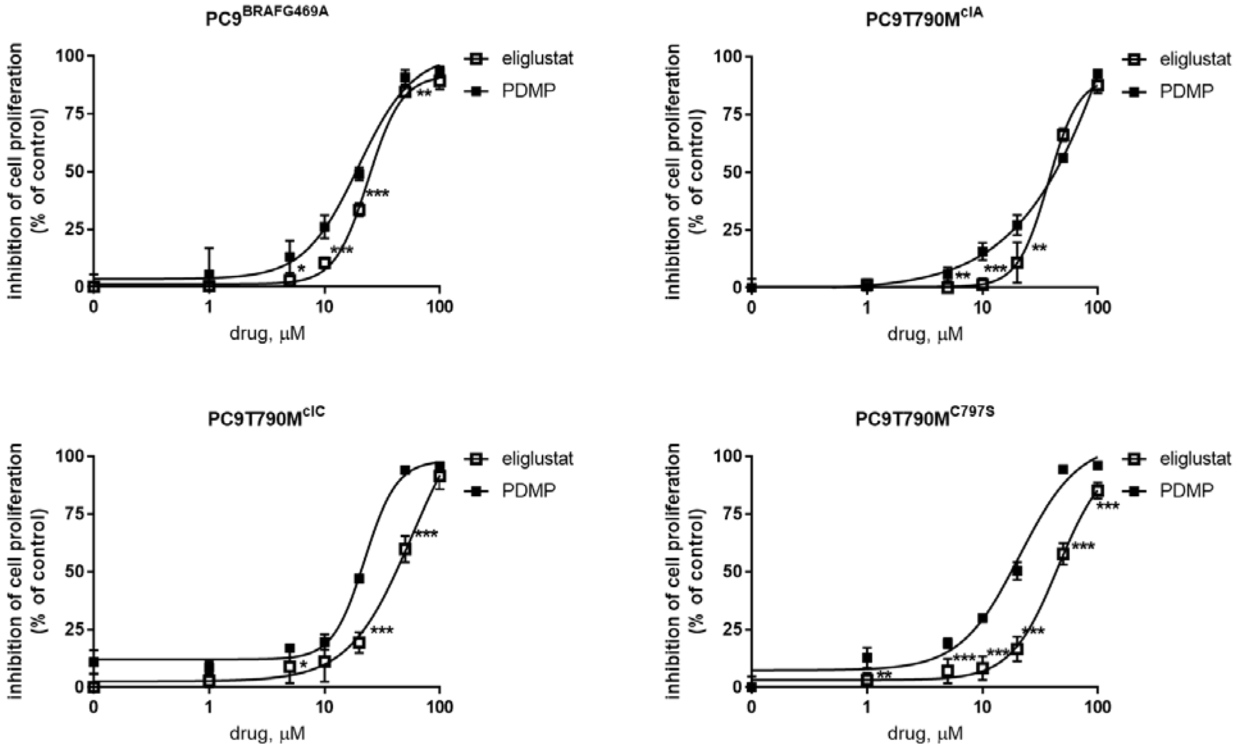

Figure S2. OR cell lines were treated with increasing concentrations of eliglustat or PDMP for 72h. Cell proliferation was assessed by MTT assay; for each cell model, the data are expressed as a percentage of inhibition vs. the corresponding untreated control cells and are means ± standard deviation (SD) of three independent determinations. * $p<0.05$, ** $p<0.01$, *** $p<0.001$ vs PDMP.

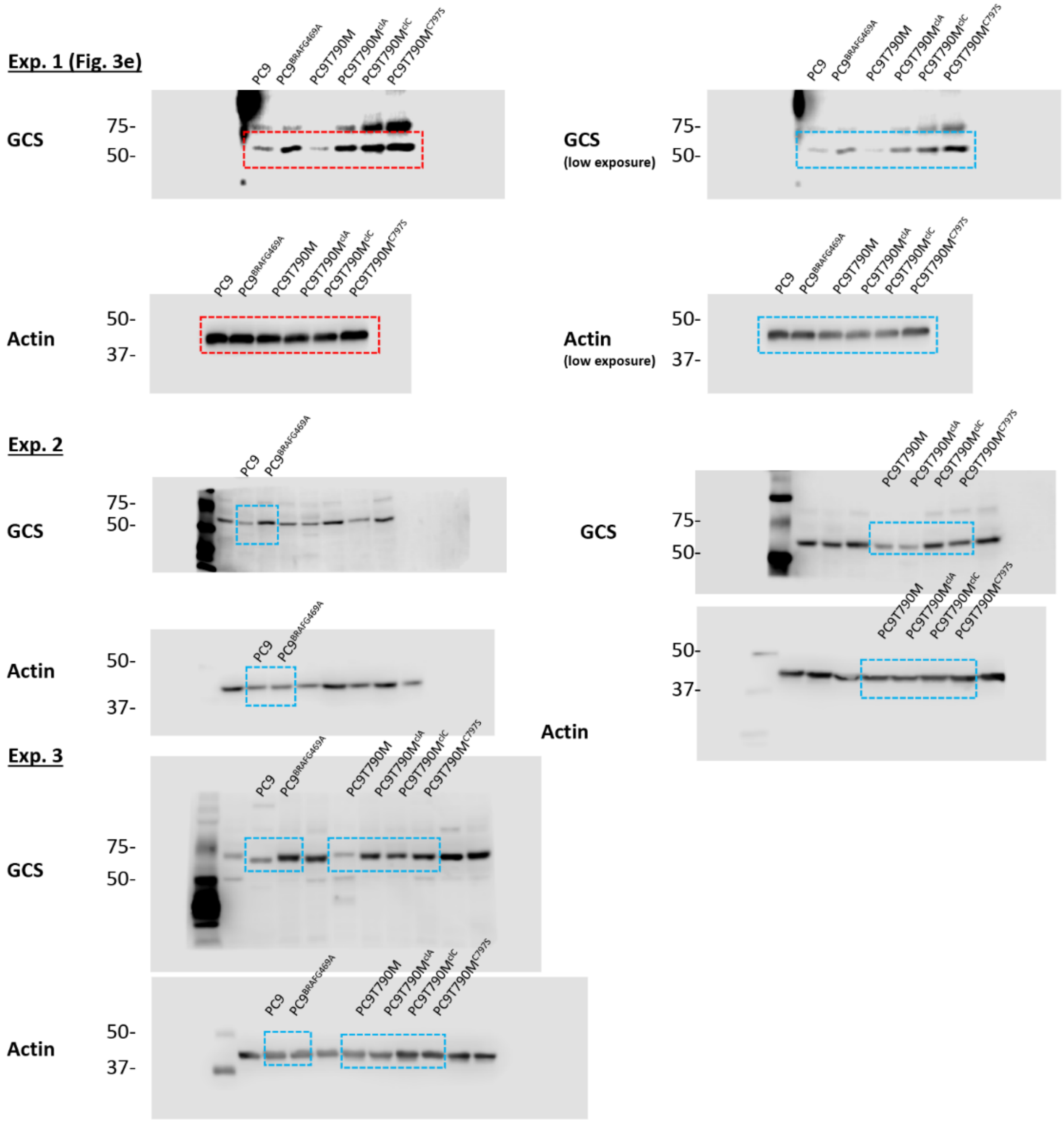

Exp. 1 (Fig. 3e)
PC9
PC9BRAFG469A
PC9T790M
PC9T790McIA
PC9T790McIC
PC9T790MC797S
GCS
75-
50-
GCS
(low exposure)
Actin
50-
37-
Actin
(low exposure)
Exp. 2
GCS
Actin
Exp. 3
GCS
Actin

Figure S3: Full original western blot images included in Figure 3E. Cropped western blot bands shown in Fig. 3e are highlighted by red boxes. Cropped western blot bands from replicates are highlighted by blue boxes. Blot membranes were cut prior to hybridisation with antibodies based on the standard band positions. As reported in the Methods Section, the chemiluminescent signal was acquired by C-DiGit® Blot Scanner. After 12 minutes of acquisition, the images with the best balance between brightness/contrast were chosen, saved in tif format with 300 dpi of resolution and cropped as reported. The identification of the bands was based on the expected molecular weight.

Exp. 1 (Fig. 6b)

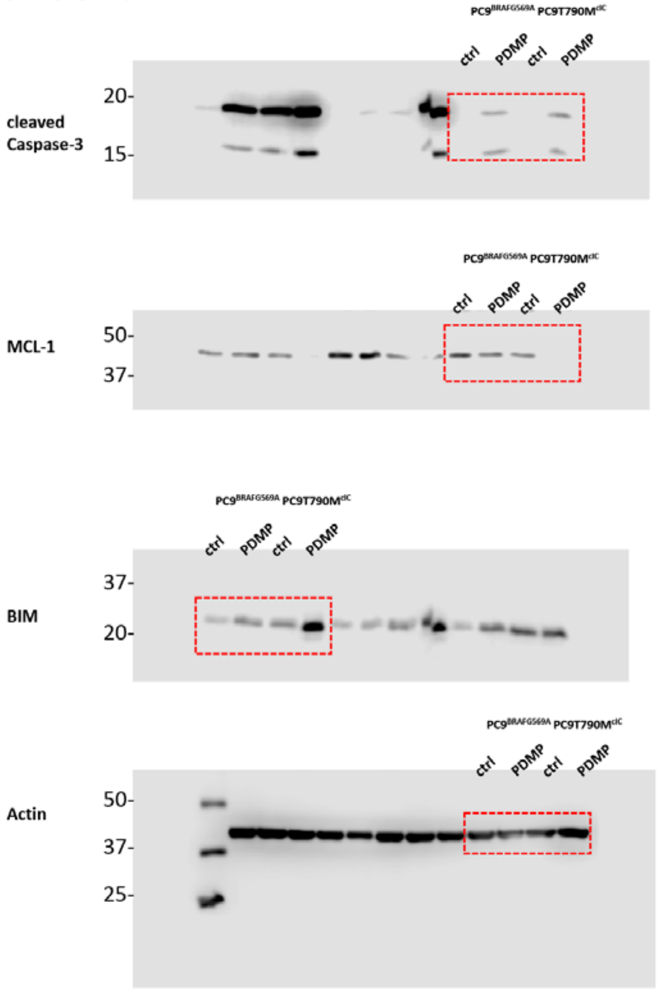

Exp. 2

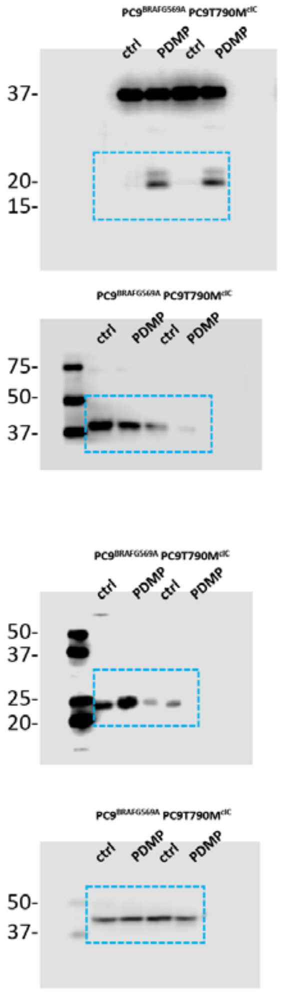

Figure S4: Full original western blot images included in Figure 6B. Cropped western blot bands shown in Fig. 6b are highlighted by red boxes. Cropped western blot bands from replicates are highlighted by blue boxes. Cropped western blot bands are highlighted by red boxes. Blot membranes were cut prior to hybridisation with antibodies based on the standard band positions. As reported in the Methods Section, the chemiluminescent signal was acquired by C-DiGit® Blot Scanner. After 12 minutes of acquisition, the images with the best balance between brightness/contrast were chosen, saved in tif format with 300 dpi of resolution and cropped as reported. The identification of the bands was based on the expected molecular weight.
